# Spatial variation of asthma rates in Los Angeles County by environmental and socioeconomic indicators

**DOI:** 10.1186/s12940-026-01298-4

**Published:** 2026-04-22

**Authors:** Parsa Khawari, Scott M. Bartell, Andrew Odegaard, Veronica M. Vieira

**Affiliations:** 1 Department of Epidemiology and Biostatistics, Joe C. Wen School of Population & Public Health, University of California Irvine, Irvine, CA USA; 2 Department of Environmental and Occupational Health, Joe C. Wen School of Population & Public Health, University of California Irvine, Irvine, CA USA

**Keywords:** Environmental epidemiology, Environmental justice, Spatial epidemiology, Asthma, Environmental degradation

## Abstract

**Background:**

Asthma is a chronic lung disease affecting nearly 1.2 million people in Los Angeles (LA) County. Asthma can be triggered and worsened by environmental pollutants. Exposure to oil/gas well operations, environmental contamination, and other forms of environmental degradation have been linked with poorer respiratory outcomes.

**Methods:**

We used Generalized Additive Models (GAMs) with a bivariate smooth of location and Quasipoisson framework to examine the relationship between location and asthma emergency department visitation (EDV) rates for census tracts in LA County (*n* = 2106). Using CalEnviroScreen scores, we predicted and mapped the rates after adjustment for environmental exposures and socioeconomic factors to determine if observed spatial patterns in asthma EDV could be explained by these known characteristics. Permutation tests were conducted to identify areas of elevated asthma EDV rates. We also calculated rate ratios (RR) and 95% confidence intervals for the indicators.

**Results:**

The rate of asthma EDVs was highest to the north of the major shipping ports in the county. Per interquartile range increase, poverty (RR: 1.29 [1.26–1.32]), percent of the population unemployed (RR: 1.02 [1.00–1.03], and solid waste site scores (RR: 1.01 [1.00–1.02]), were associated with higher asthma EDV rates. Poverty contributed the most to the observed spatial variation in asthma EDV.

**Conclusion:**

These findings can motivate future research to investigate spatial variation in asthma using higher resolution individual-level data, especially in the identified areas of elevated risk in LA County. Solid waste exposure indicators, poverty level, and unemployment explained some of the spatial variation of asthma EDV rates. Closer investigations in these geographic areas can help us better understand the disparities in exposure and risk.

**Supplementary Information:**

The online version contains supplementary material available at 10.1186/s12940-026-01298-4.

## Background

Asthma is a serious and chronic lung disease that affects nearly 25 million people in the United States and 1.2 million people in Los Angeles (LA) County [1–3]. While age-standardized rates of asthma have decreased, absolute prevalence of asthma has remained high with 2021 estimates at 260 million cases globally [4]. The inflammation, narrowing, and obstruction of airways are primary mechanisms through which asthma is triggered [5]. Exposure to traffic-related air pollution and fine particulate matter can exacerbate asthma symptoms [6, 7]. Asthma is known to vary spatially due to the clustering of communities with differing socioeconomic and demographic compositions [8], but the clustering of exposures in their physical environment may also contribute to geographic patterns of asthma. The neighborhoods near diverse green spaces, high tree clustering, and proximity to water sources were observed to experience reduced asthma emergency department visits (EDV) compared to other neighborhoods absent green and blue space, indicating that location is associated with asthma EDV risk [9, 10].

Certain environmental exposures are unique to residents of LA county. These exposures include oil and gas well operations, solid waste sites, and contamination cleanup sites that are spatially concentrated in areas of the county and may contribute to variations in asthma EDV. Unfortunately, residents are often in close proximity to multiple environmental hazards resulting in a cumulative burden of exposure that, in combination with socioeconomic co-stressors, could increase susceptibility to asthma exacerbations. The objective of this study was to explore the spatial variation of asthma and determine the contribution of environmental hazards and community sociodemographic factors to the geographic patterns observed.

LA County provides a valuable opportunity to examine the combined effects of cumulative exposures and social stressors on asthma in this impacted area. Located in one of the world’s richest oil-producing areas, the Los Angeles Basin [11], thousands of oil and gas wells have operated in Los Angeles since the late 1800s [12, 13]. Historically, oil/gas well operations have been linked to discriminatory social and environmental policies, further perpetuating the present problem of cumulative burdens. Studies and accounts have highlighted unfair risk assessment practices by the Home Owners’ Loan Corporation (HOCL) and the subsequent redlining of certain areas in LA county [14] with an increased presence of oil/gas wells [15]. Oil/gas production emits methane, hexane, benzene, and other volatile organic compounds [16–19] and has been linked with poorer respiratory and cardiovascular health outcomes, including blood pressure, wheezing symptoms, and lung function [20, 21]. Additionally, a study conducted in LA County used CalEnviroScreen (CES) scores to contextualize vulnerability in their study and showed that vulnerable communities were at higher odds of exposure to oil/gas development, signaling environmental justice inequities [22]. This study also identified disparities in oil/gas development, CES pollution burden score, and proportion of Black residents. The cumulative burden of these factors can lead to worsening health outcomes for people disproportionately exposed to both environmental and social stressors [23, 24]. This unique interplay between socioeconomic disparities and environmental hazards prompts further spatial investigation. While the geographic clustering of asthma EDVs has been established, it is important to further investigate this spatial relationship with environmental and social stressors [8].

Proximity to environmental contamination cleanup sites, i.e., Superfund and State Response, and other solid waste sites have also been linked to various health issues. Superfund sites are locations where uncontrolled hazardous waste threatens human health. These sites are managed by the Environmental Protection Agency. A study on the Ringwood Mines/Landfill Superfund Site in New Jersey found a strong association between Superfund Site exposure and the prevalence of respiratory outcomes, namely bronchitis and asthma [25]. Highlighting inequalities in exposures, this study also found that Native Americans were nearly 14 times more likely than their non-Native American counterparts to exposed to Superfund sites [25]. A national study on the location of Superfund sites determined that contaminated areas contained a higher presence of minority populations [26]. This association was further supported by a Long Island, NY study that found inequalities in the demographic distribution of those living near state and Superfund cleanup sites [27]. A cohort study of births near the New Bedford Harbor Superfund site found that, in conjunction with prenatal exposures to asthma risk factors, proximity to a Superfund site was associated with asthma-related clinical care encounters in children [28]. A study in New York State examined the association between respiratory issues and proximity to solid waste sites, finding an increased risk of asthma-related hospitalization and other respiratory diseases [29]. Studies have also found disproportionate correlations between the presence of hazardous waste sites and the racial/ethnic composition in the study areas [30, 31].

Asthma is also associated with an area’s income level; those in lower-income neighborhoods are disproportionately burdened by asthma [8, 32, 33]. The extent of this burden is also expressed through the differences in the severity of asthmatic outcomes [33]. Studies have highlighted increased financial burden because of asthma, associations between socioeconomic status and asthma, and existing disparities in asthma prevalence in different neighborhoods in urban settings [34–36]. Geographic differences in socioeconomic factors may also contribute to spatial variation of asthma.

To our knowledge, there have not been any studies in LA County examining whether environmental degradation is associated with asthma at a neighborhood level, or how much of the existing spatial variation of asthma emergency department visits (EDV) can be explained through environmental degradation and socioeconomic status. To address this research gap, we utilized the CalEnviroScreen 4.0 dataset from the California Office of Environmental Health Hazard Assessment to investigate the spatial association between asthma EDVs, environmental degradation, and socioeconomic status [37].

## Methods

### Health outcomes data

The objective of this study was to examine the spatial distribution of asthma EDVs throughout LA County census tracts and assess the contribution of environmental and socioeconomic indicators to any observed geographic variation. Released in 2021, CalEnviroScreen 4.0 [37] is a tool that employs environmental, health, and socioeconomic variables to identify vulnerable communities. CalEnviroScreen uses these indicators to create component and overall scores for the U.S. Census Bureau’s 2010 Census Tract boundaries, so that the differences between communities can be mapped and compared. In this ecological study, we utilized data for 2,343 LA county census tracts, with an approximate population of 10 million people, from CalEnviroScreen 4.0. Census tracts with no population (*n* = 16) or missing SES values (*n* = 86), were excluded. Additionally, we excluded census tracts in the northern part of LA county (*n* = 129) with low population density due to the San Gabriel Mountains [38, 39]. These census tracts were excluded since they exhibited biased behavior due to the lack of data. This is also known as “edge effects” [39]. The final analysis included 2,106 census tracts with a study population of approximately 9.2 million people. The 2019 American Community Survey was used for population sizes.

CalEnviroScreen 4.0 provided asthma EDV rates per 10,000 population. These rates were averaged from 2015 to 2017. The environmental tool sourced the rates from the State of California, Office of Statewide Health Planning and Development’s Emergency Department and Patient Discharge Datasets. ICD-9 and ICD-10 codes were used to identify EDV with a principal diagnosis of asthma. Two different classifications were used due to the replacement of ICD-9 codes in 2015. Asthma EDV rates were established at the zip-code level. These rates were reapportioned to the census tract level. Details regarding these methods are found in the CalEnviroScreen 4.0 report [37]. Count data was obtained by multiplying the provided asthma rates with the population of each census tract (Figure S1).

### Potential risk factors

#### Solid waste sites

A Solid Waste Site (SWS) is a landfill that accepts solid waste for disposal. Closed, Illegal, or Abandoned Sites (CIA), composting sites, waste tire sites, and scrap metal recyclers are subsets of solid waste landfills and were all included in CalEnviroScreen’s final SWS score. In CalEnviroScreen 4.0, Solid Waste Sites and Facilities scores are generated from data provided by CalRecycle and the Department of Toxic Substances Control (DTSC) [40–42]. CalRecycle data (2021) included data such as the site’s status (open or closed) and operational volume [37]. DTSC data (2017–2019) included information for scrap metal recycling sites. The other components of the score were the proximity of a site to the nearest populated census block, site type, and violation incurred. For example, a solid waste landfill with high operational volume would contribute more weight to the score than a closed solid waste disposal site. Additionally, sites with recorded instances of non-compliance with regulatory or safety measures were assigned higher weights. This data, in conjunction with the proximity of a site to the nearest populated census block, provided weighted scores that were aggregated to the census tract level (Figure S2).

#### Cleanup sites

Using data from the DTSC’s 2021 EnviroStor Cleanup Sites Database, a similar method was used by CalEnviroScreen 4.0 to create a score for Cleanup sites. CalEnviroScreen provides a weighing matrix that assigns weights to sites depending on the type of site they are and the status of the sites. For instance, an active Superfund site would weigh more than an inactive corrective actions sites. These weights are aggregated then assigned to census tracts [43]. This score also considered the proximity of a site to the nearest populated census block. Sites closest to populated census blocks, within a fixed radius, weighed more than those further away. Sites were ranked based on their type and status. Other site types included corrective action sites, school cleanup sites, and evaluation/military evaluation sites. A site’s status was also considered and ranked low, medium, and high depending on the extent of investigation or remediation necessary. Cleanup sites with a low status designation were considered complete or certified. Sites that were considered medium status needed further evaluation due to suspected contamination. Lastly, sites with the highest status designation were considered active with an ongoing investigation/remediation process. Site status was also considered high if the DTSC determined that remediation efforts or further investigation were necessary. The distribution of cleanup sites scores is presented in Figure S3.

#### Oil/gas wells

Oil/gas well exposure scores were not provided by CalEnviroScreen but were created for this analysis using similar methodologies. Unlike other risk factor variables, scores pertinent to oil wells were assigned to census tracts rather than the sites themselves. The Supplementary files explain the process in depth and provide additional justification for the selection of the distances. Data was attained via shapefiles from the County of Los Angeles Open Data and the California Department of Conservation, Geologic Energy Management Division (CalGEM) for 2020 [44, 45]. We included 6,810 oil and gas wells in the calculation of the exposure scores. Only active and idle oil/gas wells were considered because of the risks they pose to the environment. Oil/gas wells were considered active if they were continuously producing and were considered idle if they had been out of use for two or more years and were not properly plugged and abandoned. Idle wells were considered a threat since VOCs can leak and contaminate the surface or water sources [46]. Scores were assigned to census blocks based on the status of an oil well and the oil well’s distance to a populated census block. These scores were aggregated to generate a final score for a census tract (Figure S4). A detailed description of calculating the total score for each census tract can be found in the Supplemental Information. The creation of the oil and gas well scores was done using ArcGIS Pro 3.4.3 [47].

#### Socioeconomic indices

CalEnviroScreen provides socioeconomic data for each census tract in California. These indices include poverty, unemployment, education level, linguistic isolation, and housing burden. Spearman’s correlation test showed high collinearity between our SES variables so only poverty and unemployment were selected to prevent multicollinearity. CalEnviroScreen used a poverty metric represented by the percentage of the population living at 200% below the federal poverty line (FPL) and chose 200% due to the high cost of living in California [37]. Unemployment was quantified as the percentage of the population over 16 years of age not employed but seeking employment and are available to work. Both indices were provided by the U.S Census Bureau’s American Community Survey 5-year estimate from 2015 to 2019. Census tracts with missing estimates were excluded from the analysis (*n* = 86). Figures S5 and S6 show the distribution of these socioeconomic indices in the study area.

### Statistical analysis

#### Generalized additive models

Generalized Additive Models (GAMs) were used to examine the non-linear relationship between location and asthma EDVs. GAMs are similar to parametric regression models but include an additional bivariable smooth term to account for the X and Y dimensions of location [48].

In this study, we used a Quasipoisson model with a bivariable smooth of the longitude and latitude of LA County census tracts [48].

$$\mathbf{Log}\,\left[\mathbf{E}\left( \mathbf{X, Y}\right)\right]=\mathbf{S}\left(\mathbf{X, Y}\right)+\mathbf{log}\left(\mathbf{Population}\right)+\boldsymbol{\gamma^{\prime}}\mathbf{Z}+\mathbf{S}(\mathbf{W}),$$

where asthma counts are predicted at the centroids of each census tract by smoothing location (S(X, Y)). Offsetting by population considers the varying population sizes across census tracts. γ′Z and S(W) represent the linear and non-linear terms in our model, respectively. We examined the scores for linearity using locally weighted regression smooth to identify non-linearity. We used quasipoisson models to allow for overdispersion of the data, which often occurs in geographic studies [49]. With quasipoisson methods, we assume a more general functional form of the variance function where the variance-mean relationship is constant rather than equal and not accounting for overdispersion can lead to the underestimation of our standard errors [50–52].

#### Smoothing

Smoothing is a nonparametric technique that allows for the inclusion of nonlinear terms in our model. Smooth terms do not assume a strict relationship between predictors and the outcome variable and allow us to estimate a trend in the data [38]. For this study, the locally weighted regression smooth or loess method was used for its flexibility in determining the optimal smoothing parameter, referred to as a span size, when locally fitting our data [38]. Selecting a smaller span size may introduce greater variance while reducing bias. Large span sizes will increase bias but reduce variability [39].

#### Disease mapping and importance of location

Maps of EDV rates were created using a grid based on the latitude and longitude of the dataset and predicting the GAM model on the new grid data. A map of crude EDV rates (per 10,000 residents) was first created to examine the spatial pattern without any environmental or sociodemographic predictors. To examine spatial patterns in EDV rates after accounting for environmental or sociodemographic predictors, maps of single-covariate adjusted EDV rates were created by setting the covariate of interest to its median value in the prediction grid data and including only the single covariate in addition to longitude and latitude and a constant population of 10,000. Maps of fully adjusted EDV rates were created by predicting the fully adjusted model on the complete grid data, including all covariates and setting them at their median values. The range of the crude asthma EDV rates was used as a standard range for the color scale across all maps to facilitate visual comparisons. Asthma EDV rates were also predicted for varying levels of solid waste scores because this variable demonstrated potential non-linearity (FIGURE S7). These maps were created by using the 5th and 95th percentile values of the solid waste site score in the prediction grid, while keeping other covariates at their median values.

To test the significance of the association between disease and location, we used a permutation test of the deviance between models with and without the smoothed location term. The locations of the census tracts were permuted 999 times, models were refit with each permuted dataset, and the deviance of the true model was ranked in the distribution to determine a global p-value for the significance of location [39]. Predictions from each permuted model were also ranked to identify areas of lower and elevated asthma rates by using the lower and upper 2.5% of the distribution generated by the permutation test. All statistical analyses were conducted in R using the MapGAM package [53, 54].

#### Sensitivity analysis

We considered additional variables (Diesel PM score, PM2.5 score, and Pesticide Score) that represent individual components of exposures from the environmental hazards described previously. While the scores for hazard sites capture multiple exposures from these sources, these exposure scores may reflect impacts from a combination of different sources. These variables are explored both in univariate and fully adjusted analyses with corresponding maps. We also conducted a univariate analysis of the overall CES 4.0 score which measures the cumulative burden from environmental factors and socioeconomic indicators together in one score.

## Results

### Descriptive statistics

A total of 49,675 asthma EDVs were included in our analysis. Across southern LA census tracts, the average rate of emergency department visits for asthma from 2015 to 2017 was 53.52 per 10,000 people. The environmental and socioeconomic variables are summarized in Table 1 by quartile. Average environmental indicator scores were 43.68, 11. 85, and 2.20 for oil/gas well exposure, cleanup sites exposure, and solid waste sites exposure, respectively. Higher scores are indicative of greater exposures. The population includes a substantial proportion that are economically burdened, with an average of 35.41% of the population living two times below the FPL and 6.15% of the population being unemployed.

**Table 1:** Characteristics of environmental and socioeconomic indices in LA County census tracts, by quartile

Risk Factors	Asthma Counts by Census Tracts			
	1st quartile	2nd quartile	3rd quartile	4th quartile
Oil/Gas Score				
Mean (SD)	39.67 (144.28)	33.77 (190.44)	59.10 (293.14)	42.23 (209.43)
Median (IQR)	0.00 (4.44)	0.00 (2.375)	0.00 (3.25)	0.00 (3.969)
Range	0.00 -1447.38	0.00–3082.88	0.00–3567.88	0.00–2660.12
Cleanup Sites Score				
Mean (SD)	7.79 (11.23)	11.70 (16.40)	13.50 (18.20)	14.42 (22.74)
Median (IQR)	3.10 (11.25)	4.50 (14.87)	5.90 (18.09)	6.375 (15.09)
Range	0.00–70.35	0.00–109.10	0.00–103.90	0.00–276.20
Solid Waste Sites Score				
Mean (SD)	1.526 (3.06)	2.075 (4.28)	2.44 (3.65)	2.727 (5.17)
Median (IQR)	0.200 (1.75)	0.500 (2.50)	1.000 (3.20)	1.000 (3.45)
Range	0.00–26.10	0.00–63.40	0.00–27.70	0.00–64.25
Poverty (%)				
Mean (SD)	24.27 (16.13)	32.58 (16.69)	39.41 (16.83)	45.42 (15.75)
Median (IQR)	18.70 (20.55)	29.50 (26.60)	38.10 (25.90)	45.90 (23.45)
Range	3.20–80.80	4.80–79.10	4.70–86.50	6.90–86.30
Unemployment(%)				
Mean (SD)	5.33 (2.95)	5.82 (2.75)	6.42 (2.85)	7.03 (3.55)
Median (IQR)	5.00 (3.5)	5.40 (3.5)	6.05 (3.68)	6.40 (4.38)
Range	0.00–31.80	0.00–17.50	0.40–20.50	1.50–35.90

*SD* standard deviation, *IQR* interquartile range

### Spatial analysis

**Fig. 1:**
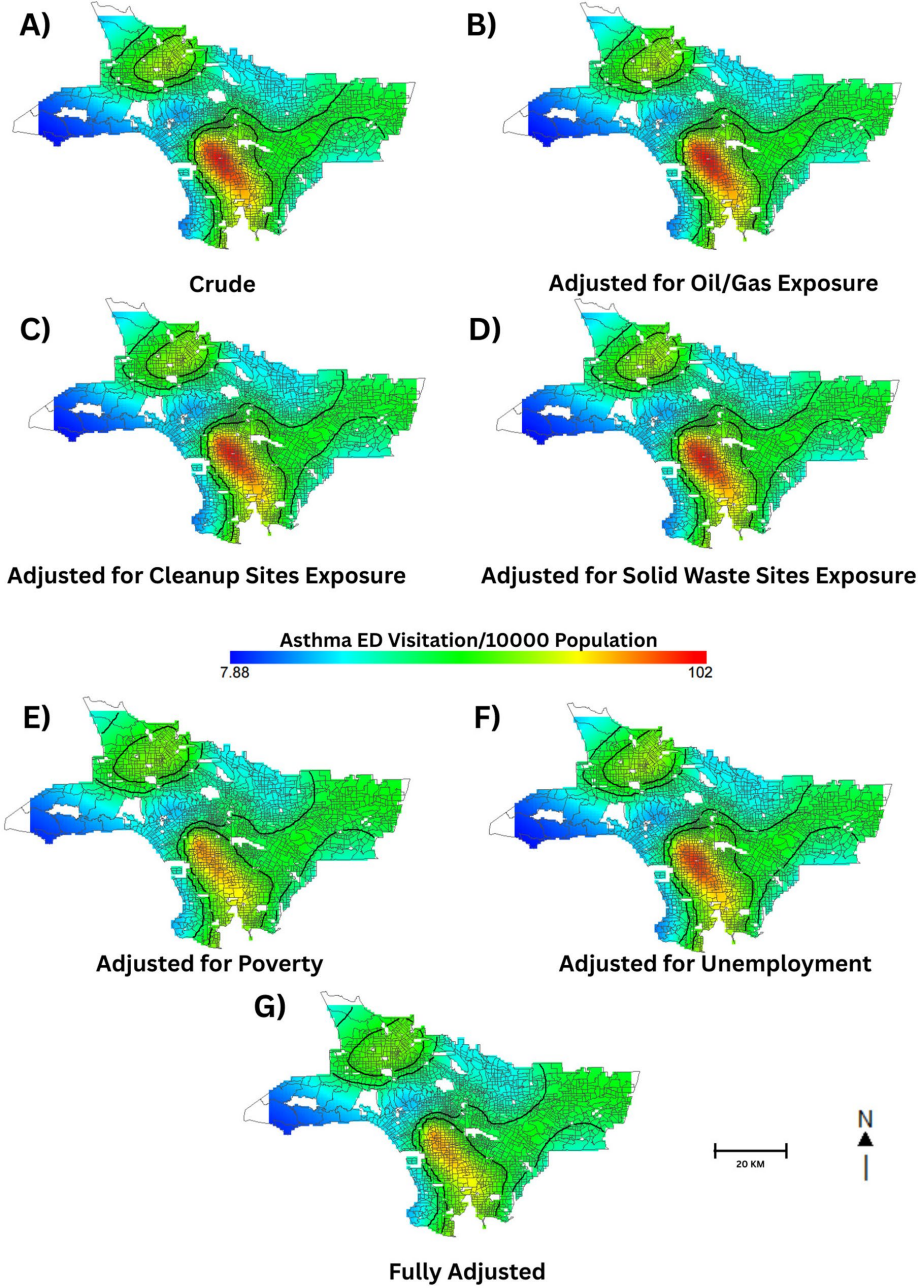
Crude, Univariate, and Fully Adjusted Maps of 2015–2017 Asthma EDV Rates, Southern LA County Census Tracts. **A** Crude Asthma EDV rates, **B** Asthma EDV rates after adjusting for oil/gas well exposure, **C** adjusted for cleanup sites exposure, **D** adjusted for solid waste sites exposure, **E** adjusted for poverty, **F** adjusted for unemployment, **G** fully adjusted. Areas of significance are denoted by black contour lines

In the crude model, location was significantly associated with rates of asthma EDVs as indicated by the permutation test global statistic (*p* < 0.001). Areas of elevated EDV rates were identified in the south (north of major shipping ports in the county) and northwest areas of the study region. Areas of significance are denoted by black contour lines (Fig. 1A). Spatial patterns did not change after adjusting for the first environmental indicator, oil/gas well exposure score (Fig. 1B). This suggests that oil/gas well exposure scores did not explain the spatial variation of asthma EDVs identified in the crude map. Spatial variation after adjusting for exposure to cleanup sites showed slight attenuation of asthma EDVs rates (Fig. 1C). Introducing solid waste site scores to the model also led to a slight shift in spatial variation when compared to the crude model where areas of significance widened to include East LA census tracts. Location remained significantly associated with asthma EDV rates even with the inclusion of our environmental indicators; furthermore, spatial patterns were consistent across models, suggesting that these indicators do not meaningfully contribute to the observed variation of asthma EDV rates (Fig. 1D). Adjusting for poverty resulted in a notable change in the spatial variation of asthma EDVs, with attenuated rates in the southern region of the study area compared to our crude model (Fig. 1E). Adjusting for the percentage of the unemployed population also explained some of the spatial variation in the southern census tract that was observed in the crude analysis (Fig. 1F). All indicators exhibited an approximately linear relationship with asthma EDV rates, although the smooth for solid waste site score did exhibit some non-linearity in part of the curve (Figure S7).

The fully adjusted model included all environmental and socioeconomic indicators. Compared to the crude and univariate models, the fully adjusted model explained more of the spatial variation of asthma EDVs in LA County census tracts (Fig. 1G). However, the contour lines around areas of increased rates and the general spatial pattern of the health outcome remained relatively the same, indicating unmeasured spatial confounding that was not captured by our environmental and socioeconomic indicators. Poverty, percent unemployment, and solid waste sites scores were significantly associated with the asthma EDV patterns observed in LA County census tracts. Asthma EDV rates were associated with an interquartile range (IQR) increase in poverty (RR: 1.29 [1.26–1.32]), percent of the population unemployed (RR: 1.02 [1.01–1.03], and solid waste site scores (RR: 1.01 [1.00–1.02]) (Table 2). Per IQR increase, oil/gas exposure scores (RR: 1.00 [1.00–1.00]), and cleanup sites scores (RR: 1.01 [1.00- 1.02]) were not associated with the asthma EDV. The fully adjusted model also resulted in a slight increase in rates in census tracts along the coast, but this was not statistically significant as these tracts fell outside the contour lines.

**Table 2:** Univariate and fully adjusted model summaries

Model	Range of Predicted Rates	Global *P*-Value	Univariate RRs (95% CI)^§^		Fully Adjusted RRs (95% CI)^§**^
Crude	7.88–102.15	*p* < 0.001		---	---
Oil/Gas Score	7.88–102.23	*p* < 0.001		1.00 (1.00–1.00)	1.00 (1.00–1.00)
Cleanup Sites Score	7.93–101.57	*p* < 0.001		1.02 ( 1.00–1.03)*	1.01 (1.00–1.02)
Solid Waste Sites Score	7.85–101.70	*p* < 0.001		1.01 (1.00–1.01)	1.01 (1.00–1.02)*
Poverty (%)	10.73–88.63	*p* < 0.001		1.30 (1.27–1.33)*	1.29 (1.26–1.32)*
Unemployment (%)	8.12–98.46	*p* < 0.001		1.06 (1.05–1.08)*	1.02 (1.00–1.03)*

§ Location is included in the model; RRs were calculated using IQRs; Best span size determined by AIC was 0.15 for all models

* Statistically Significant

**Range of predicted rates for the fully adjusted model is 10.70–87.82 and the global p-value is < 0.001

Asthma EDV rates were also predicted at varying levels of the Solid Waste Site scores. The purpose of this was to assess how the reduction or increase of this score would change asthma rates. Using the fully adjusted model, solid waste site scores were set to the 5th and 95th percentiles to predict asthma EDVs (Figure S8 & S9). At the 95th percentile, an increase in the range of predicted asthma EDV rates were observed, from 10.70 to 87.82 to 11.00–90.33 per 10,000 population. At the 5th percentile, a marginal decrease in range was observed to 10.68–87.68 per 10,000 population. The 5th and 95th percentile solid waste site scores were 0 and 9.5, respectively.

### Sensitivity analysis

Table S1 shows the univariate and fully adjusted model summaries of our models with the inclusion of Diesel PM, PM2.5, and Pesticides. We also examined CES 4.0 score in its own univariate model but did not include it in the fully adjusted model as it is a composite score that includes all the other components. Figures S10-S13 present univariate maps for these variables. The overall CES 4.0 score explained the greatest amount of spatial variation while also yielding high risk ratios, 1.54 (1.52–1.57) in the univariate analysis. Given the definition of the CES 4.0 score, a composite score that considers both pollution and socioeconomic indicators, this is expected. In the fully adjusted model, poverty continued to have the greatest impact on asthma EDV rates (RR: 1.28, [1.25, 1.31]). All other variables had either null or small, but protective, associations (Table S1).

## Discussion

This study investigated the spatial variation of asthma EDV rates in LA County. Through the utilization of GAMs, we included location as a risk factor modeled by the bivariate smoothing of longitude and latitude [48, 55]. Doing so, we were able to identify areas of higher and lower asthma rates and adjusted for risk factors to determine their contribution to the identified spatial variation. Elevated areas of asthma EDV rates were present in the northwest and southern areas of LA county. Notably, increased rates were observed north of major shipping ports. Several studies have explored the movement of goods through LA county’s shipping ports, including the large presence of diesel vehicles near port communities and infrastructure connected with the port such as freight yards [56, 57]. A closer investigation into port communities may yield valuable insight regarding the disparities in asthma EDVs. Solid waste sites, poverty, and unemployment were associated with higher asthma EDV rates, with socioeconomic indicators explaining much of the spatial variation observed with the health outcome.

Our results are aligned with other studies that examined the relationship of asthma with socioeconomic status and vulnerable populations [32–36, 58, 59]. This study also supports findings that examined proximity to waste sites and respiratory issues [20, 21]. While the effect estimate for solid waste sites was relatively small compared to that of poverty, greater utilization of solid waste sites, inappropriate processing of waste, and the creation of additional waste sites can introduce increased environmental risks in these areas. In addition, the locations and impacts of solid waste sites are amenable to different regulations and policy interventions compared to poverty, so it may be more practicable to achieve reductions in EDV by targeting this variable for intervention, even though poverty has larger impacts. It is also important to note that the solid waste site metric is a composite score that takes into account different types of facilities. It would be valuable to further investigate these individual components, so that we can better understand which sites pose the greatest health risks.

After adjusting for our environmental and socioeconomic covariates, the spatial variation of asthma EDV rates remained statistically significant, and the boundaries of areas of increased rates did not change. Oil/gas well and cleanup site exposure scores did not explain the spatial variation of asthma. Exploring the independent effects of oil and gas and accounting for production may improve our ability to observe an association. Literature has shown that health disparities exist among those exposed to oil/gas well operations, particularly respiratory function [13, 22]. Oil/gas well operations emit harmful exposures that are linked with poor respiratory function [19, 21]. Disaggregating oil wells from gas wells and considering oil/gas well output (barrels per day) in our score development can improve our scoring methodology and better capture the burden these operations pose.

Using census tract data, we may not be able to capture environmental exposure pathways on which hazards act. This may be due to the study design’s limitations, rather than the inherent impacts of each environmental or sociodemographic factor on EDV. Ecological studies and their findings are strictly representative of the population and are notoriously difficult to adjust fully for the effects of confounding variables [60]. Estimates and inferences generated from this study cannot be applied to individuals. Proximity to oil/gas wells are also unique exposures due to the geologic nature of oil fields and where these resources are being collected [11]. Capturing these exposures and their association to asthma EDVs may require a study at a finer spatial resolution. Spatial analyses using individual level data can provide more insight into the risk factors that contribute to the spatial variation of a health outcome [39, 55, 61]. It is also important to consider that EDVs do not describe the full extent of asthma’s burden and underestimate the true count of asthma cases. Those who practice disease management and have access to preventative care are less likely to report to an ED for asthma related issues, which may explain why poverty is such a strong explanatory variable for the observed spatial patterns of EDV in this study. However, asthma EDVs are a good marker for the largest component of socioeconomic burden of the disease and capture vulnerable populations who lack access to routine care [37]. It is important to note that several census tracts had missing data. In CalEnviroScreen’s calculations, missingness depends on standard errors and relative standard errors. In the event of small population sizes, census tracts may be excluded. However, small populations may be indicative of resource-limited settings. These census tracts should still be investigated to better understand the limitations and burden on the presiding population. Furthermore, investigation into unmeasured confounders like pollen count, average temperature, wind patterns, and other quantifications of the ambient environment could also provide insight into the identified spatial disparities.

These findings highlight the importance of managing waste sites and enforcing operational guidelines to improve public health. Additionally, policies aimed at reducing or restricting the operational volumes of solid waste sites closer to populated areas can also reduce disparities in asthma EDV rates. The complex nature of socioeconomic determinants and their relationship with health disparities requires a multi-level approach. This study introduces evidence of elevated asthma EDV rates in different areas in LA county and demonstrates that both environmental and socioeconomic factors can explain these results. Using this knowledge, efforts can be concentrated into closely investigating these areas to better understand the pathways that these risk factors act upon.

## Conclusion

This study found areas of elevated asthma EDV rates among LA County census tracts that were partially explained by solid waste site scores, poverty, and unemployment. We observed no associations with oil/gas well scores and cleanup site scores. After adjustment for important environmental and socioeconomic covariates, we found that spatial variation remained statistically significant, indicating the presence of unmeasured confounding. Our findings suggest that some of the burden of asthma EDVs may be relieved by addressing solid waste sites and other instances of environmental degradation. This study introduces evidence that prompts closer investigation into these communities with elevated risk. Future studies into these areas at a finer scale are essential in minimizing health disparities and protecting vulnerable neighborhoods.

## Supplementary Information

Supplementary Material 1.

Supplementary Material 2.

Supplementary Material 3.

Supplementary Material 4.

Supplementary Material 5.

Supplementary Material 6.

Supplementary Material 7.

Supplementary Material 8.

Supplementary Material 9.

Supplementary Material 10.

Supplementary Material 11.

Supplementary Material 12.

## Data Availability

All data supporting the findings of this study are available within the paper and its Supplementary Information.
