# Supplementary material for: Spatial variation of asthma rates in Los Angeles County by environmental and socioeconomic indicators
Source: Environ Health. 2026 Apr 22;25:48. doi: 10.1186/s12940-026-01298-4 (PMC13238009; doi:10.1186/s12940-026-01298-4)
Supplement: Supplementary file 11 — Supplementary Material 11. [file 12940_2026_1298_MOESM11_ESM.docx]

**Supplementary Information for :**

**Investigating the Spatial Variation of Asthma in Los Angeles County Utilizing CalEnviroScreen 4.0 Environmental and Socioeconomic Indicators**

Authors

Parsa Khawari^1^, Scott M. Bartell^1,2^, Andrew Odegaard^1^, Veronica M. Vieira^2^

1. Department of Epidemiology and Biostatistics and 2. Department of Environmental and Occupational Health, Joe C. Wen School of Population & Public Health, University of California, Irvine, CA

Parsa Khawari [pkhawari@uci.edu](mailto:pkhawari@uci.edu)

**Figure S1:** Asthma Emergency Department Visit Counts for Southern LA County Census Tracts.


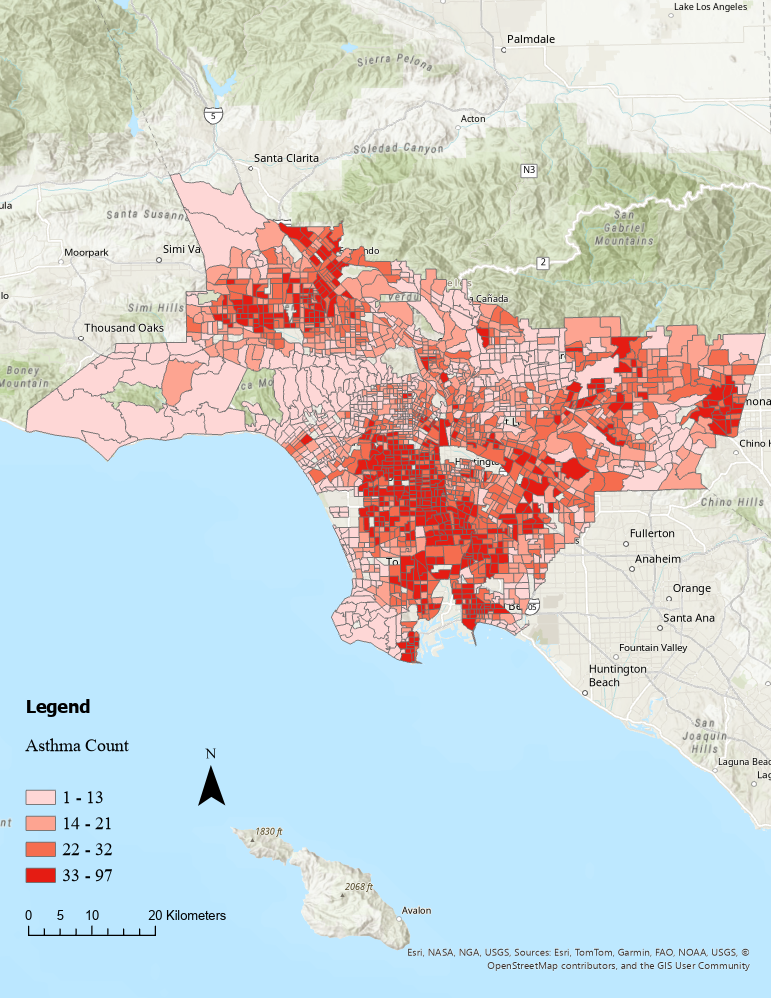


**Figure S2:** Solid Waste Sites Exposure Score for Southern LA County Census Tracts, by equal interval. Presented using individual component scores.


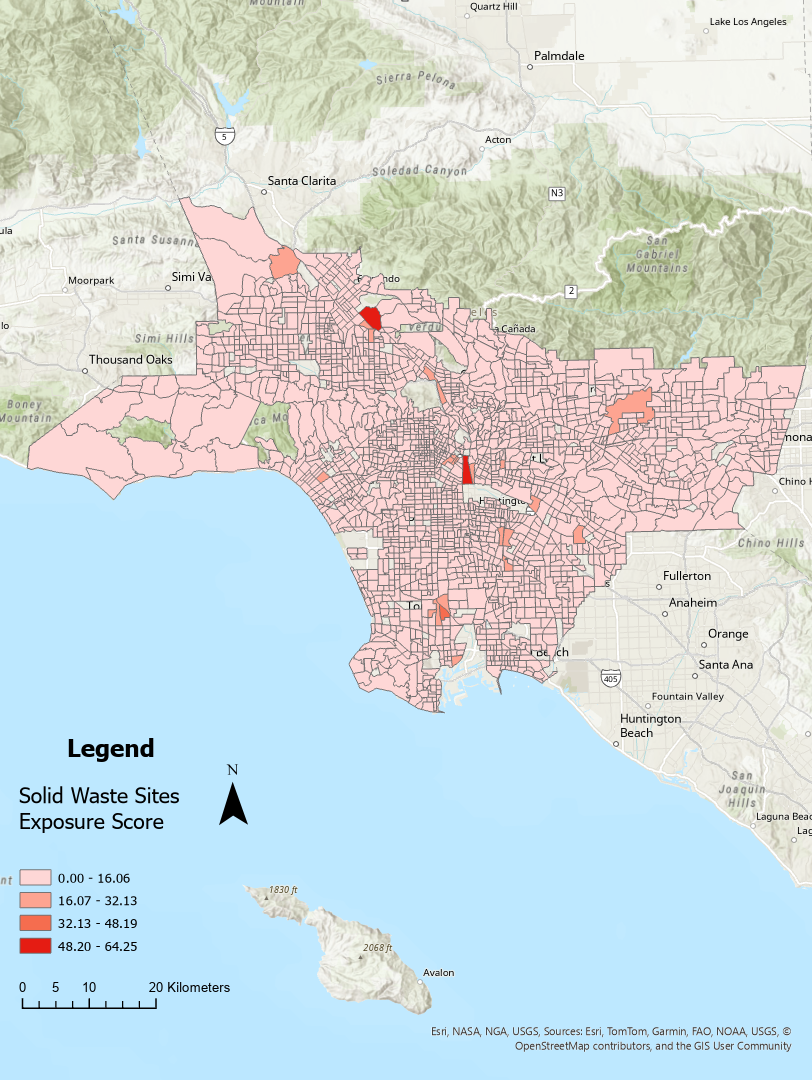


**Figure S3:** Cleanup Sites Exposure Score for Southern LA County Census Tracts, by equal interval. Presented using individual component scores.


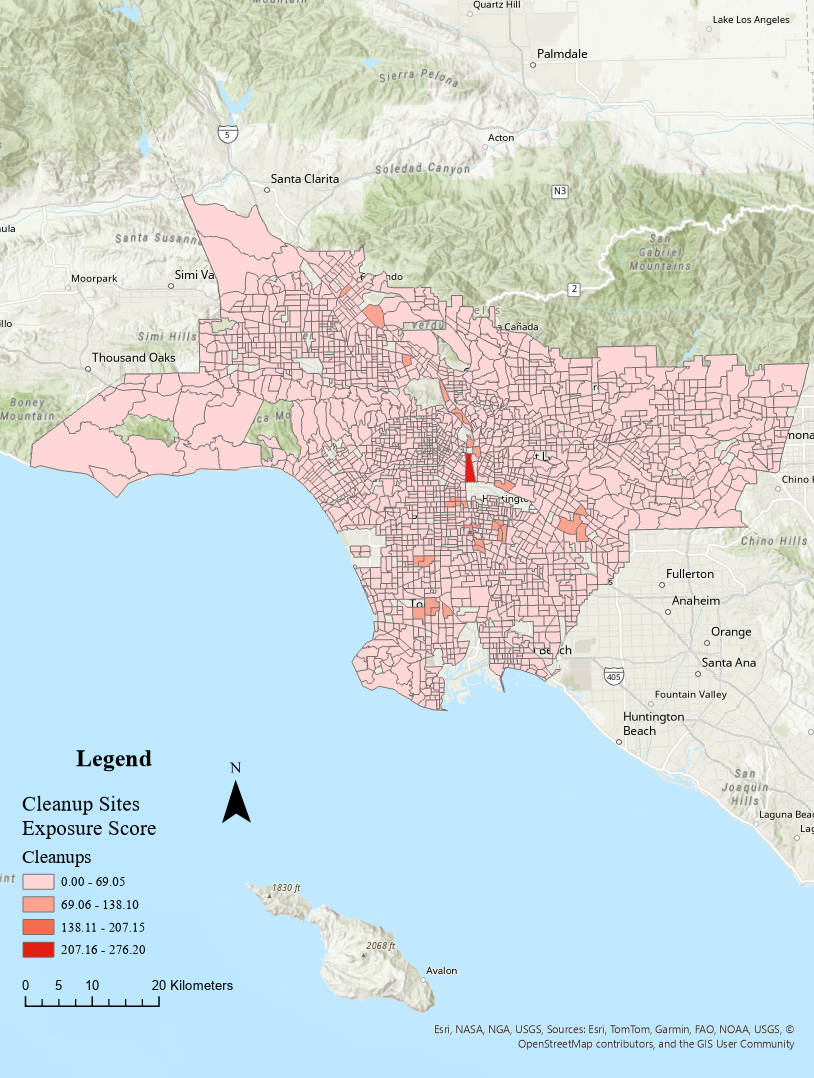


**Figure S4:** Oil/Gas Well Exposure Score for Southern LA County Census Tracts, by equal interval. Presented using individual component scores.


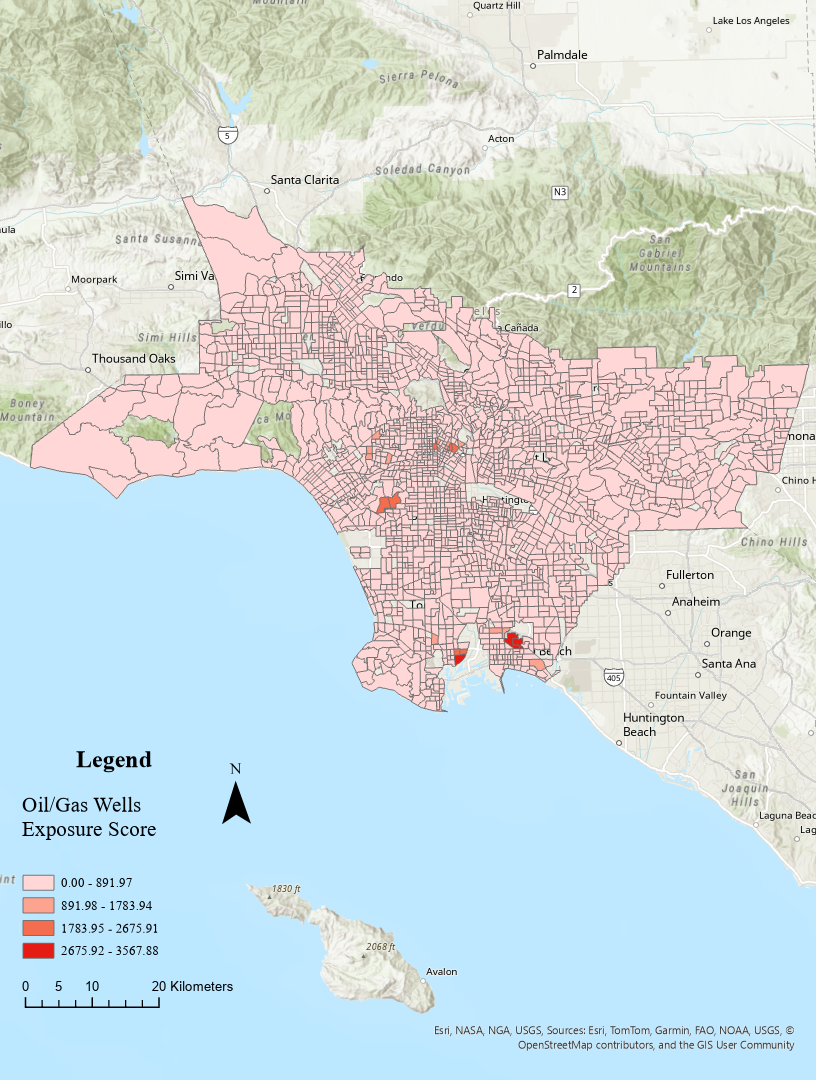


**Figure S5:** Percent of the population 2x under the federal poverty line for Southern LA County Census Tracts, by equal interval.


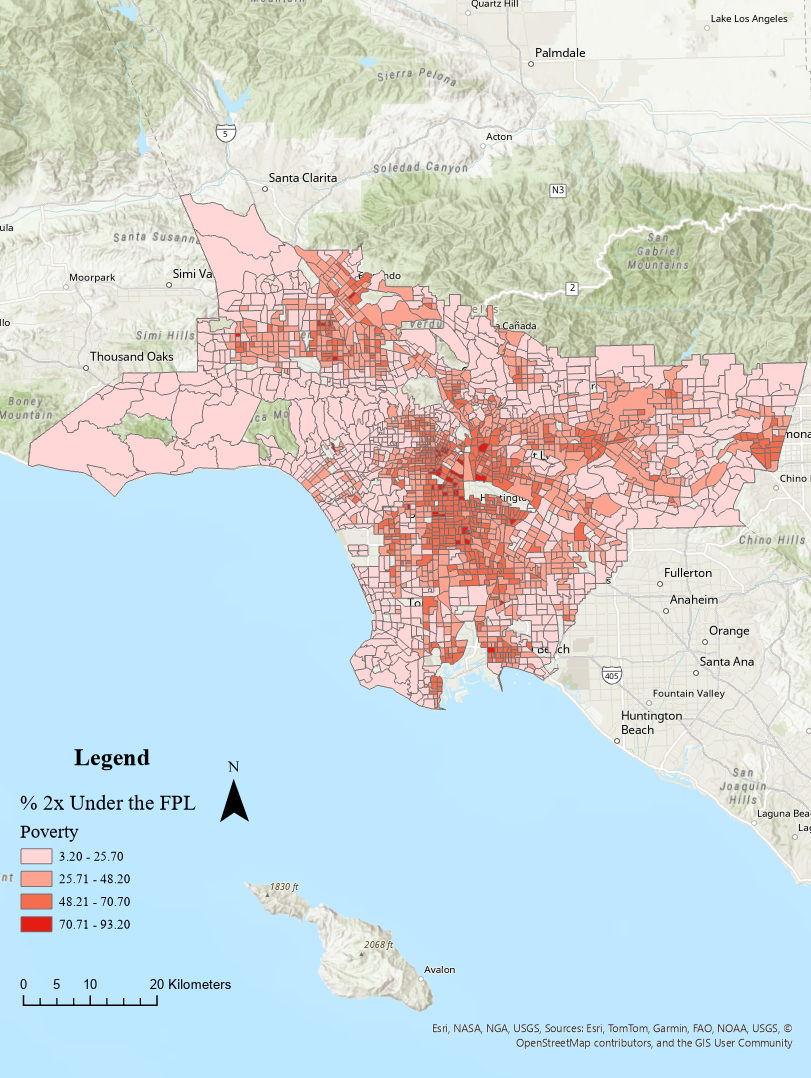


**Figure S6:** Percentage of the population unemployed for Southern LA County Census Tracts, by equal interval.


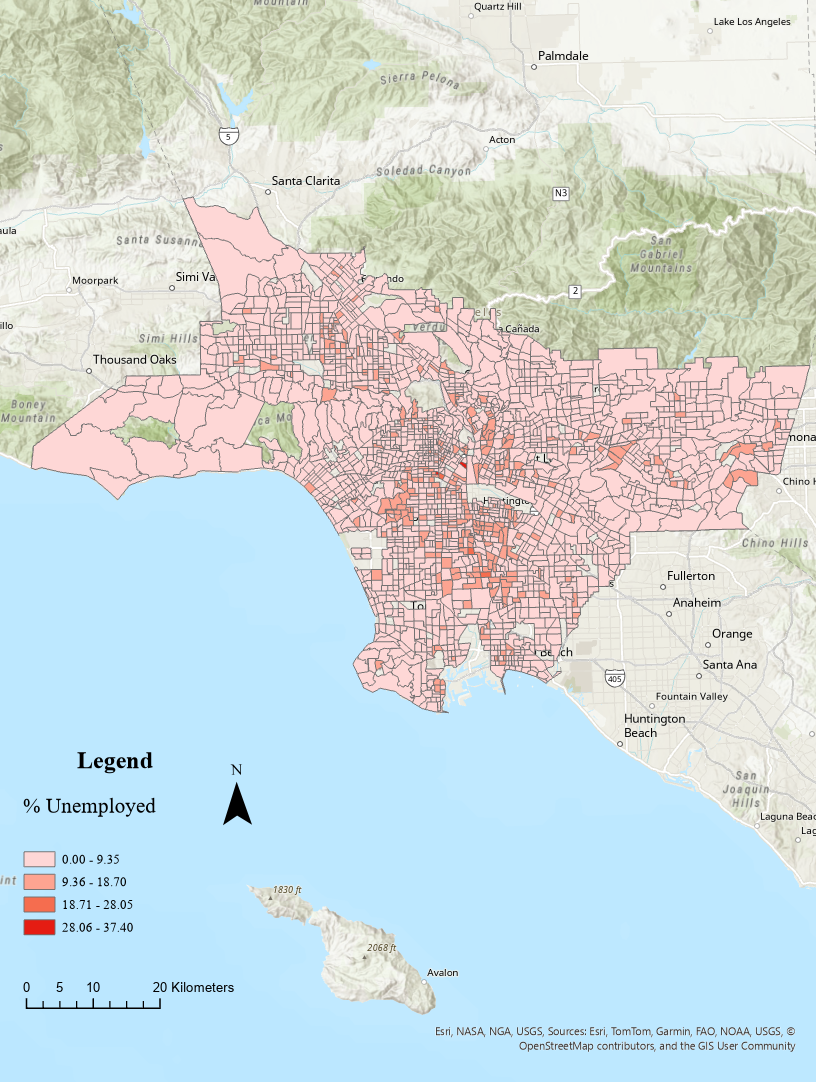


**Figure S7**: Secondary Analysis of Solid Waste Site Exposures


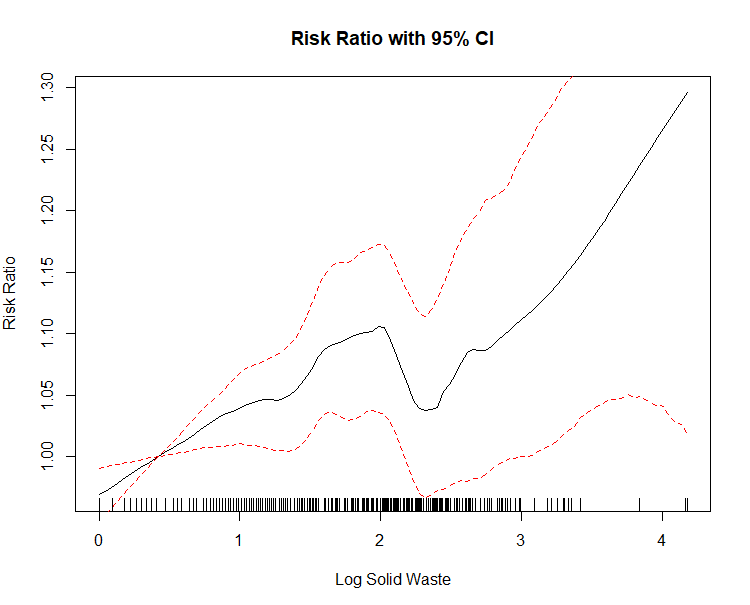


**Figure S8**: Map of Solid Waste Site Exposures scores at the 95th percentile*


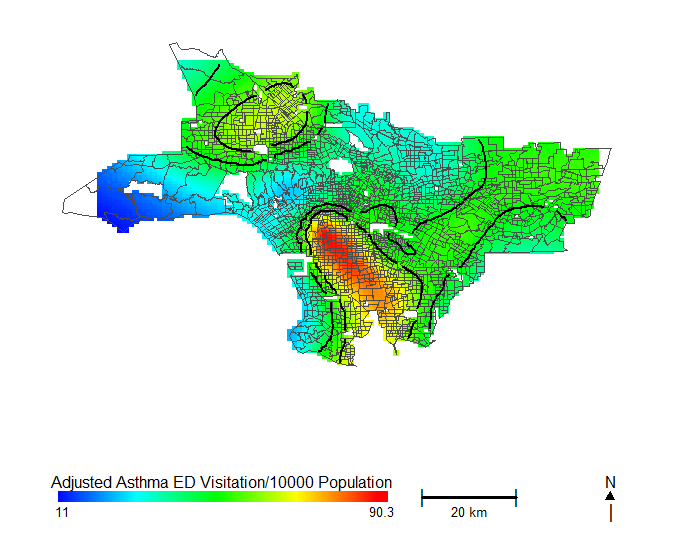


*While holding all other Covariates at their median values

**Figure S9:** Map of Solid Waste Site Exposures scores at the 5th percentile*


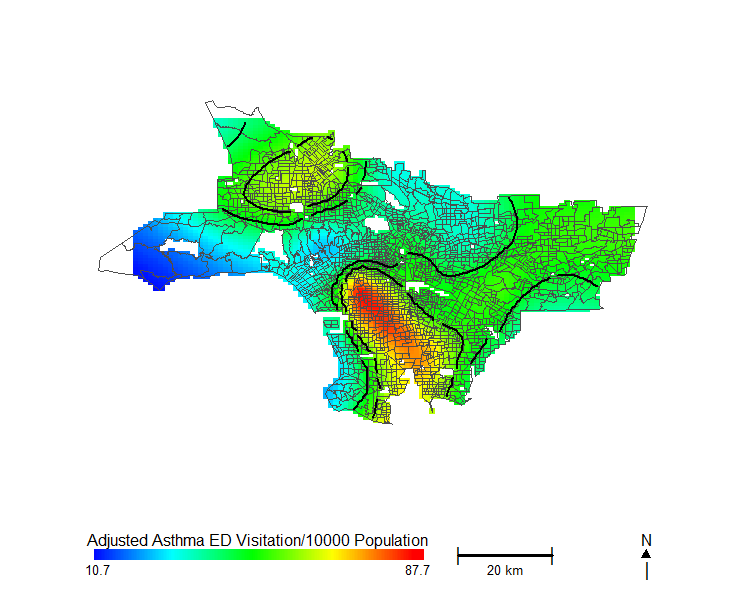


*While holding all other Covariates at their median values

**Sensitivity Analyses:**

**Figure S10**: Map of 2015 - 2017 Asthma EDV Rates, Southern LA County Census Tracts, adjusted for CES 4.0 scores


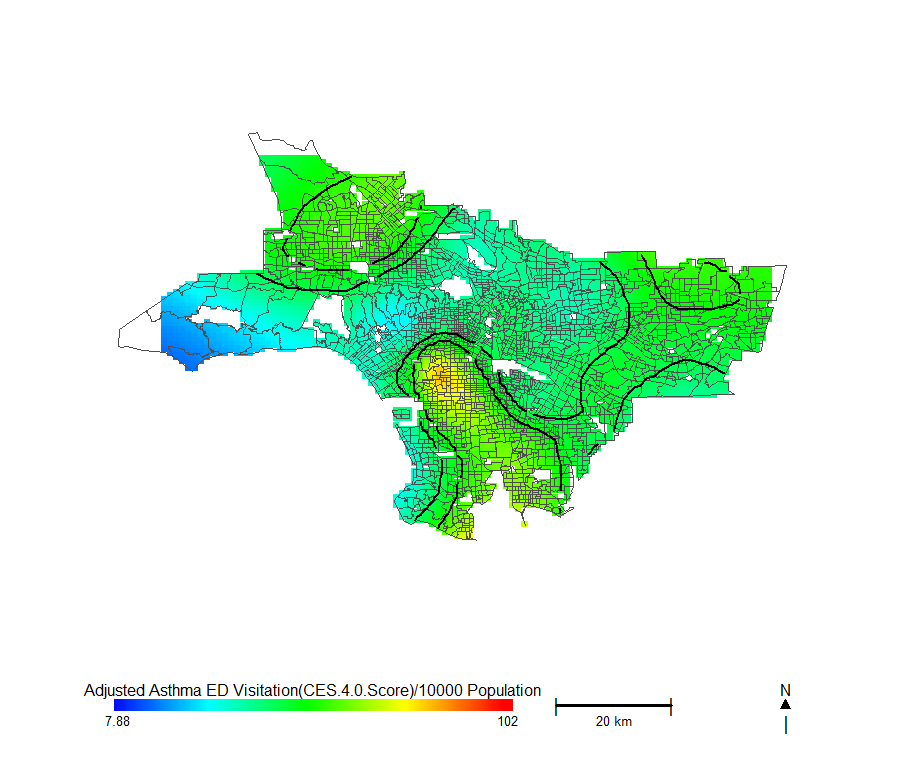


**Figure S11**: Map of 2015 - 2017 Asthma EDV Rates, Southern LA County Census Tracts, adjusted for Diesel PM scores


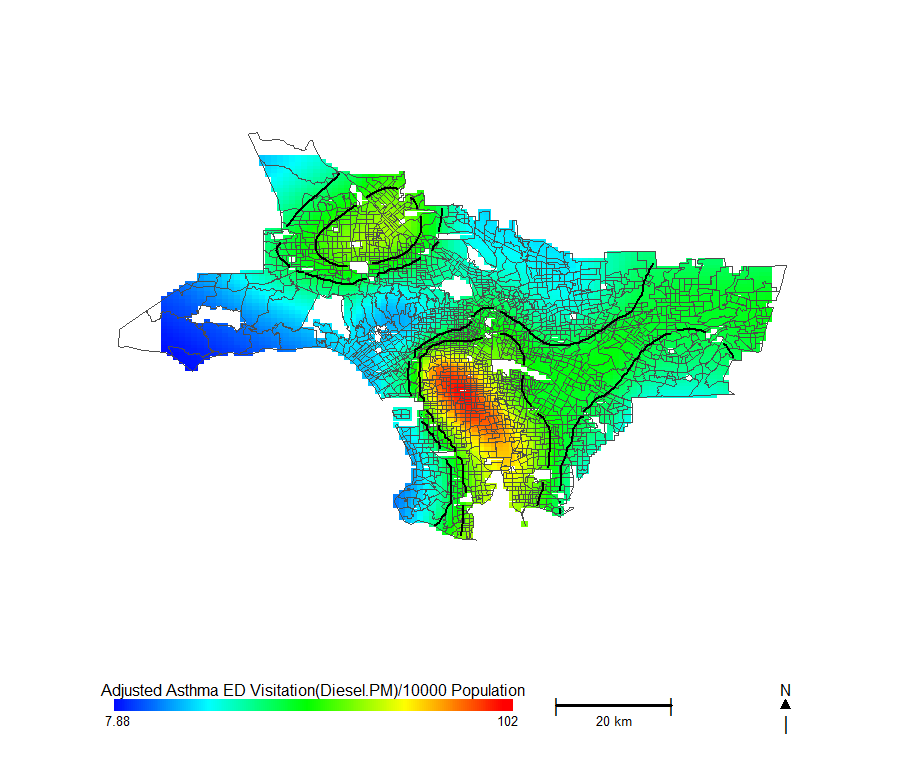


**Figure S12**: Map of 2015 - 2017 Asthma EDV Rates, Southern LA County Census Tracts, adjusted for PM2.5 scores


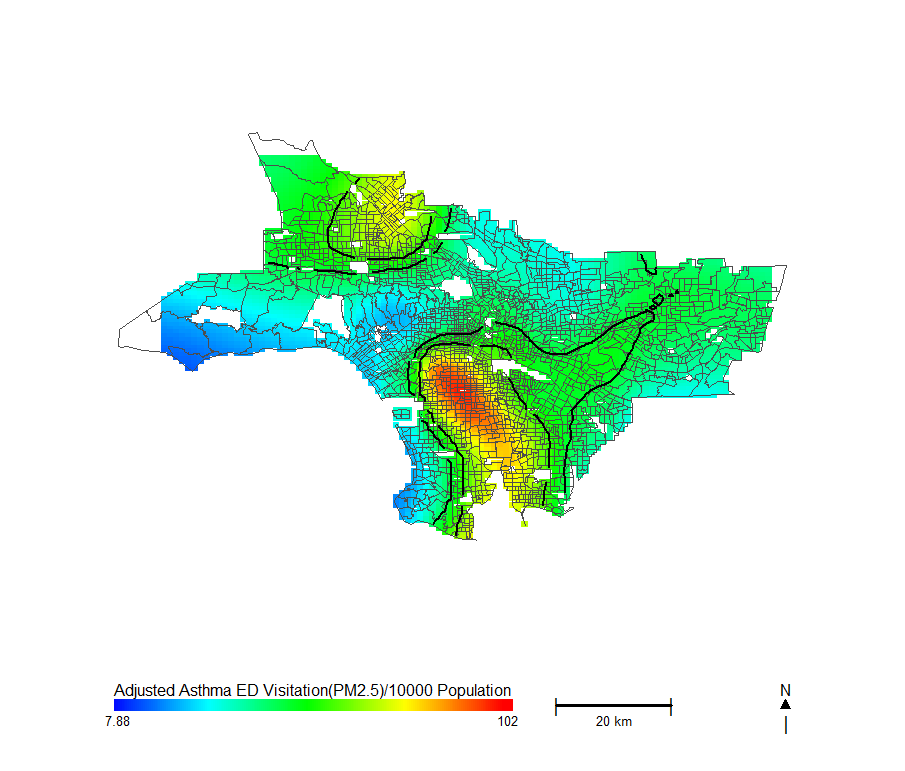


**Figure S13**: Map of 2015 - 2017 Asthma EDV Rates, Southern LA County Census Tracts, adjusted for Pesticide scores


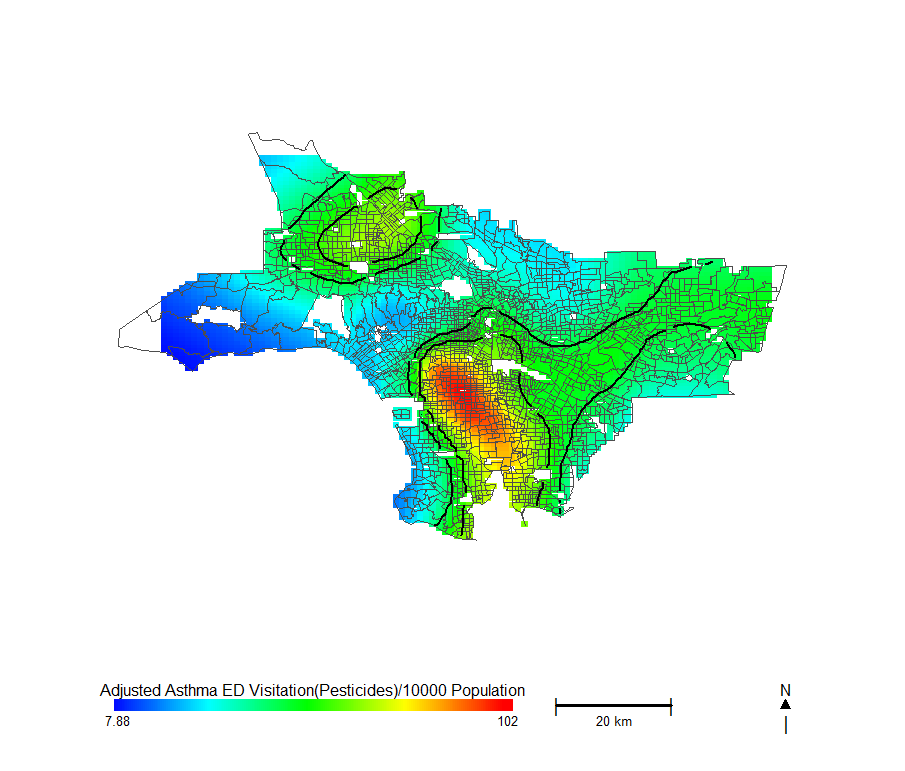


Table S1: Univariate and Fully Adjusted Model Summaries

| **Model** | **Range of Predicted Rates** | **Global P-Value** | **Univariate RRs (95% CI)^§^** | | **Fully Adjusted RRs (95% CI)^§**^** |
| --- | --- | --- | --- | --- | --- |
| **Crude** | **7.88 - 102.15** | **p < 0.001** | | **---** | **---** |
| **CES 4.0 Score** | **17.00 - 83.95** | **p < 0.001** | | **1.54 (1.52 -1.57)*** |  |
| **Oil/Gas Score** | **7.88 - 102.23** | **p < 0.001** | | **1.00 (1.00 - 1.00)** | **1.00 (1.00 - 1.00)** |
| **Cleanup Sites Score** | **7.93 - 101.57** | **p < 0.001** | | **1.02 (1.00 - 1.03)*** | **1.01 (0.99 - 1.03)** |
| **Solid Waste Sites Score** | **7.85 - 101.70** | **p < 0.001** | | **1.01 (1.00 - 1.01)** | **1.01 (1.00 - 1.02)*** |
| **Poverty (%)** | **10.73 - 88.63** | **p < 0.001** | | **1.30 (1.27 - 1.33)*** | **1.28 (1.25 - 1.31)*** |
| **Unemployment (%)** | **8.12 - 98.46** | **p < 0.001** | | **1.06 (1.05 - 1.08)*** | **1.02 (1.00 - 1.03)*** |
| **Diesel PM** | **7.95- 101.84** | **p < 0.001** | | **1.01 (1.00 - 1.02)*** | **0.99 (0.97 - 1.00)*** |
| **PM2.5** | **14.90- 99.72** | **p < 0.001** | | **1.08 (1.07-1.09)*** | **1.06 (1.05 - 1.07)*** |
| **Pesticides** | **7.88-102.20** | **p < 0.001** | | **0.99 (0.99-1.00)** | **0.99 (0.99 - 1.00)*** |

**§ Location is included in the model; RRs were calculated using IQRs**

*** Statistically Significant**

****Range of predicted rates for the fully adjusted model is 21.31 - 87.35 and the global p-value is <0.001**

**Best span size determined by AIC was 0.15 for all models**

**Oil/Gas Well Score Methodologies**

To calculate oil/gas exposure scores per census tracts, we started with census blocks that were populated. Various weights were applied based on an oil/gas wells distance to the nearest populated census block and oil/gas well type. Only active and idle oil/gas wells were used because of their potential to contaminate air, soil, and groundwater (1). Distances that were selected in the final analysis were 250m, 500m, and 1000m. We modeled the weights using literature that explores the association of oil/gas well exposure and poorer health outcomes. Distances in these studies covered exposures within 1 kilometer (2, 3). Thus, 1000m was the furthest distance considered when weighing the scores.

Six shapefiles were created to capture the oil/gas wells: 3 shapefiles for active oil/gas wells at the chosen distances and 3 shapefiles for idle oil/gas wells at the chosen distances. These shapefiles were intersected with a shapefile of populated census blocks.

1. A screenshot of the attribute table of the intersection between active oil well buffers and populated census tracts. The “ORIG_FID” indicates the ID of the oil/gas well. “BUFF_DIST” indicates the buffer of that oil/gas well that intersects with a populated census block.


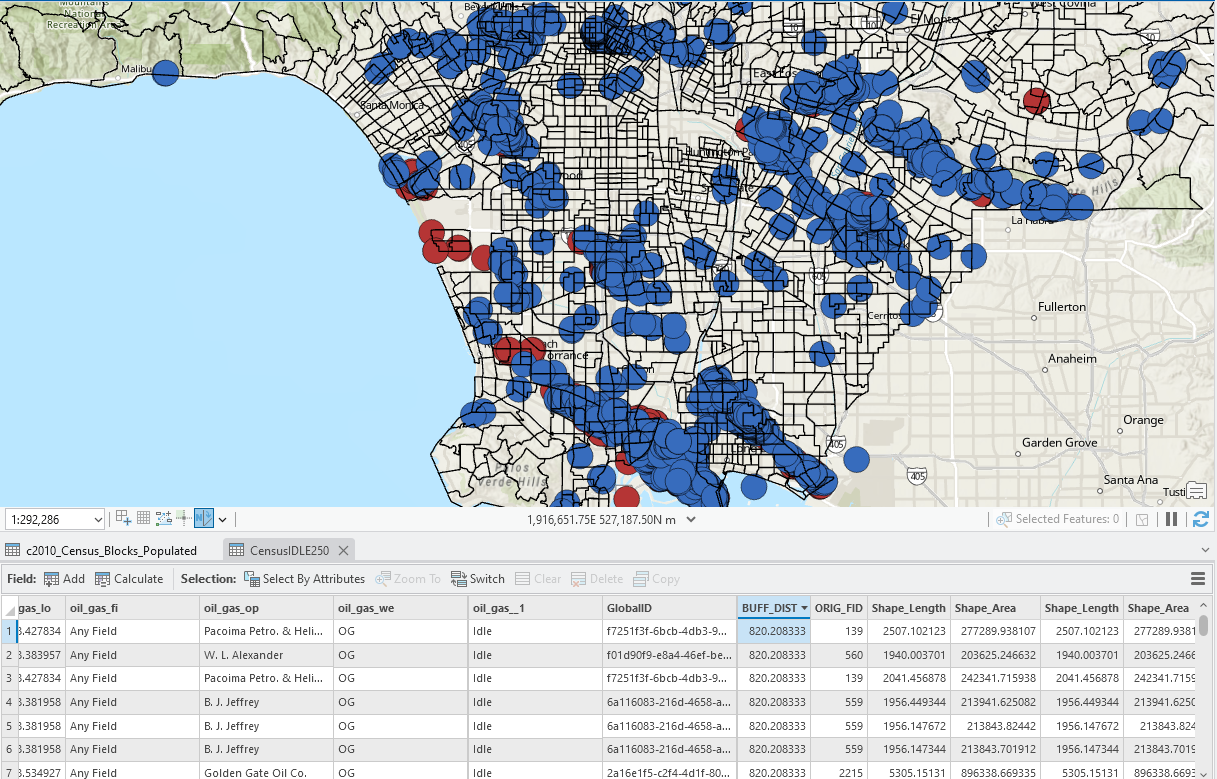


We did not want to count the same oil/gas well multiple times if multiple buffers from the same well intersected census block. To account for this, a census block was scored based on the first buffer that intersected with it.

1. The screenshot below shows the 3 different ranges (BUFF_DIST values) resulting when a single oil/gas well (ORIG_FID 3347) is intersected by the same census block (CTCB10 9800221043). It also shows an oil/gas well (ORIG_FID 3346) intersecting different census blocks with different buffer distances. If we aggregated oil/gas well scores at this point, the same well would contribute multiple times to the same census tract. We wanted to capture only the closest well buffer that intersects with any given census block.


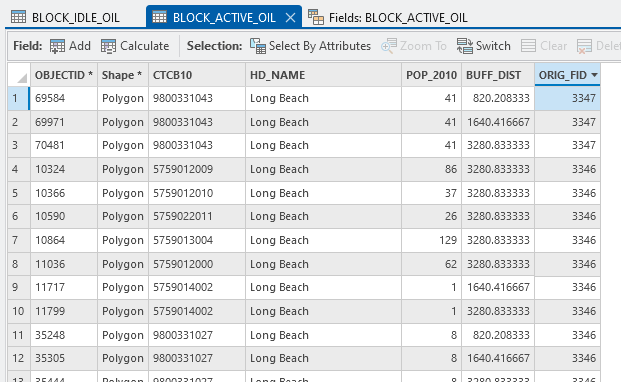


To address this, we summarized the buffer distance fields to calculate statistics for the census blocks and wells. This created census block and well pairings where each block and intersecting well were only included once at the smallest buffer distance.

1. This screenshot shows the resulting unique block-well pairings at the smallest intersecting buffer distance.


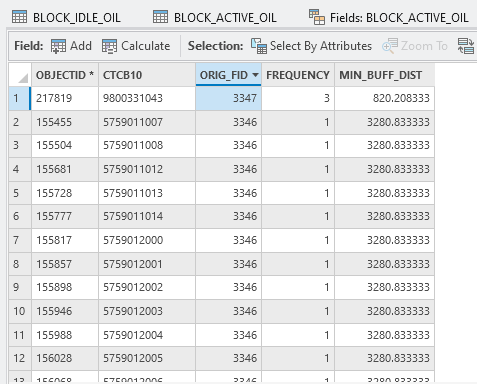


To assign the scores, we created a new field and assigned a score based on distance. Active oil/gas wells within 250 meters of a census block contributed a weight of 1 to that census block. An oil/gas well at 500 meters would contribute 0.50 weight and an oil/gas well at 1000 meters would contribute 0.25 weight. These values were all halved for idle oil/gas wells.

1. This screenshot displays the attribute table of active oil wells and their distance-based scores.


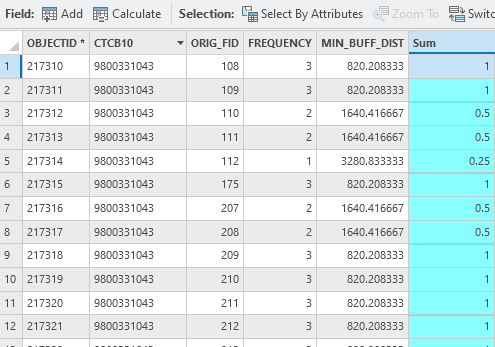


We scored each block-well pair. We then summed the individual scores within each populated census block to determine the final oil/gas well scores. The process was repeated with idle oil/gas wells, as they were weighed differently.

1. This screenshot displays the attribute table of census blocks and their respective active oil well scores.


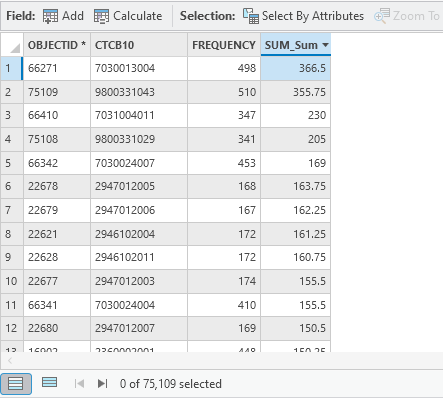


1. Active and idle scores were then joined to the original populated census block shapefile and summed for a score that captures both well types for every census block.


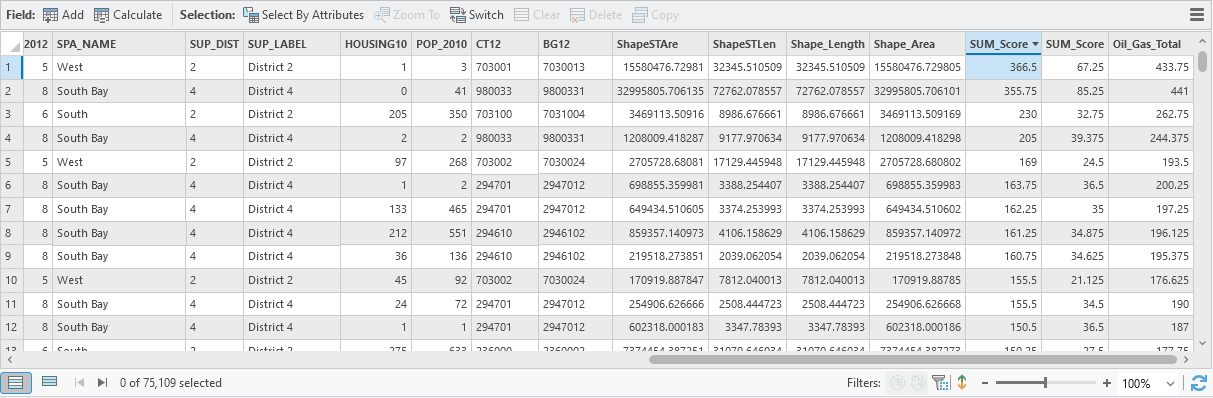


1. The final oil/gas well score was calculated by summing all the scores for each census tract.


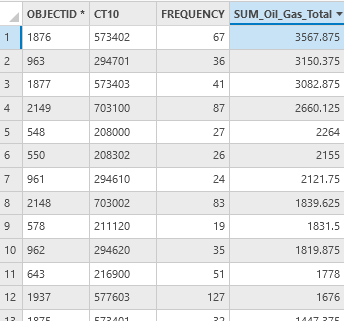


**References**

1. California Department of Conservation. Idle well program [Internet]. Available from: https://www.conservation.ca.gov/calgem/idle_well
2. Johnston JE, Enebish T, Eckel SP, Navarro S, Shamasunder B. Respiratory health, pulmonary function and local engagement in urban communities near oil development. Environmental Research [Internet]. 2021 Mar 29;197:111088. Available from: https://pubmed.ncbi.nlm.nih.gov/33794173/
3. Chan M, Shamasunder B, Johnston JE. Social and environmental stressors of urban oil and gas facilities in Los Angeles County, California, 2020. American Journal of Public Health [Internet]. 2023 Sep 6;113(11):1182–90. Available from: https://ajph.aphapublications.org/doi/10.2105/AJPH.2023.307360
