# Supplementary material for: Spatial variation of asthma rates in Los Angeles County by environmental and socioeconomic indicators
Source: Environ Health. 2026 Apr 22;25:48. doi: 10.1186/s12940-026-01298-4 (PMC13238009; doi:10.1186/s12940-026-01298-4)
Supplement: Supplementary file 12 — Supplementary Material 12. [file 12940_2026_1298_MOESM12_ESM.docx]

library(sf)

library(car)

library(MapGAM)

library(dplyr)

library(ggplot2)

setwd('F:/THESIS/IMPORTANT')

datamap=st_read(dsn='SOUTH_LA_FINAL.shp', layer='SOUTH_LA_FINAL')

practice <- read.csv ('F:/THESIS/IMPORTANT/SOUTH_LA_FINAL.csv')

practice <- read.csv ('F:/THESIS/IMPORTANT/SOUTH_LA_FINAL.csv')

plot(st_geometry(datamap))

points(practice$Centroid_X,practice$Centroid_Y,pch=19)

points(practice$Centroid_X,practice$Centroid_Y,pch=19)

span.aic = matrix(ncol=1,nrow=17)

for (S in 3:19) {fit=gam(Asth_Count~lo(Centroid_X,Centroid_Y,span=S*0.05)+offset(log(Population)), family=poisson, data=practice)

span.aic[S-2,1]=fit$aic}# STORE THE AIC FROM THE MODEL IN THE MATRIX

# DETERMINE THE SPAN WITH THE MINIMUM AIC AND PRINT RESULTS

sp=(which(span.aic[,1]==min(span.aic[,1]))+2)*0.05

cat(paste("The span that minimizes the AIC is ", sp,".", sep=""), fill=TRUE)

#Creating Prediction Grid

# CREATE GRID OF POINTS FOR PREDICTIONS USING predgrid FUNCTION IN

# MapGAM LIBRARY

newgrid=predgrid(X=practice$Centroid_X, Y=practice$Centroid_Y, map=datamap)

Centroid_X = newgrid[,1]; Centroid_Y=newgrid[,2]

Population=10000 # ADD POPULATION OFFSET TO GRID

data.grid=data.frame(cbind(Centroid_X,Centroid_Y,Population))

plot(st_geometry(datamap))

points(data.grid[,1:2],pch=19)

gridlen=length(data.grid[,1])

gridlen

m.crd=gam(Asth_Count~lo(Centroid_X,Centroid_Y,span=0.15)+offset(log(Population)),

family=poisson, data=practice)

summary (m.crd)

m.crd.0=gam(Asth_Count~ offset(log(Population)), family=quasipoisson, data=practice)

anova(m.crd.0,m.crd,test='Chisq')

coords=practice[,3:4] # DATA COODINATES

m.data=practice # CREATE NEW DATASET

devrank=matrix(ncol=1, nrow=1000, 0)# DEVIANCE - GLOBAL

devrank[1]=anova(m.crd.0, m.crd, test="Chisq")[2,4]

permresults=matrix(ncol=1000, nrow=gridlen, 0) # POINTWISE - LOCAL

permresults[,1]=predict(m.crd, data.grid)

#PERMUTATING DATA

i=j=2

while (j<1001) {

index=sample(length(m.data$Asth_Count), replace=F)

m.data[,3:4]=coords[index,]

m.gam=gam(Asth_Count~ lo(Centroid_X,Centroid_Y, span=0.15)+offset(log(Population)), family=poisson, data = m.data)

devrank[i]=anova(m.crd.0, m.gam, test="Chi")[2,4] # NEW MODEL DEVIANCE

permresults[,i]=predict(m.gam, data.grid) # CREATE DISTRIBUTION

if (j%%100==0) print(i)

i=j=j+1 }

hist(permresults)

observed_value <- devrank[1]

library(ggplot2)

# Create a data frame for plotting

perm_df <- data.frame(

Deviance = devrank[-1], # Exclude observed value

Type = "Permutation"

)

observed_df <- data.frame(

Deviance = observed_value,

Type = "Observed"

)

# Combine data

plot_data <- rbind(perm_df, observed_df)

# Plot

ggplot(plot_data, aes(x = Deviance, fill = Type)) +

geom_histogram(data = perm_df, aes(y = ..density..), binwidth = 0.5, alpha = 0.6, color = "black") +

geom_vline(data = observed_df, aes(xintercept = Deviance), color = "red", linetype = "dashed", size = 1.2) +

labs(title = "Permutation Distribution with Observed Deviance",

x = "Deviance",

y = "Density") +

theme_minimal() +

theme(legend.position = "none")

# P-VALUE FOR DEVIANCE GLOBAL STATISTIC

p.crd=(1000-rank(devrank)[1])/1000 # THE HIGHEST DEVIANCE RANKS 1000

p.crd

#RANKING LOG RISK AT EACH POINT

permrank=matrix(ncol=1000, nrow=gridlen, 0)

for (k in 1:gridlen) {

permrank[k,]=rank(permresults[k,])}

#CREATE LIST FOR MAPPING IN R

data.fit.c=list(grid=data.grid[,1:2], fit=exp(predict(m.crd,data.grid)), pointwise.permt=permrank[,1]/1000)

range(data.fit.c$fit)

#MAP RESULTS

map1 <- colormap(data.fit.c, as_Spatial(datamap), contours="permrank", contours.lwd=2, legend.name="Crude Asthma ED Visitation/10000 Population")

#COVARIATE 1///////////////////////////////////////////////////////////////////////////////////////////////////////////////

span.aic = matrix(ncol=1,nrow=17)

for (S in 3:19) {fit=gam(Asth_Count~lo(Centroid_X,Centroid_Y,span=S*0.05)+offset(log(Population)) + SUM_OILGAS, family=poisson, data=practice)

span.aic[S-2,1]=fit$aic}# STORE THE AIC FROM THE MODEL IN THE MATRIX

sp=(which(span.aic[,1]==min(span.aic[,1]))+2)*0.05

cat(paste("The span that minimizes the AIC is ", sp,".", sep=""), fill=TRUE)

#FITTING OPTIMAL SPAN

3.5 - 0

#3.5

exp(-5.080e-05 *3.5)

#0.9998222

#Upper 95%

exp(-5.080e-05*3.5+2.756e-05*3.5*1.96)

#1.000011

#Lower 95%

exp(-5.080e-05*3.5-2.756e-05*3.5*1.96)

#0.9996332

#ADDING COVARIATES TO GRID

data.grid$SUM_OILGAS=median(practice$SUM_OILGAS, na.rm = TRUE)

#HYPOTHESIS TESTING

coords=practice[,3:4] # DATA COODINATES

m.data=practice # CREATE NEW DATASET

devrank=matrix(ncol=1, nrow=1000, 0)# DEVIANCE - GLOBAL

devrank[1]=anova(m.cov1.0, m.cov1, test="Chi")[2,4]

permresults=matrix(ncol=1000, nrow=gridlen, 0) # POINTWISE - LOCAL

permresults[,1]=predict(m.cov1, data.grid)

anova(m.cov1.0, m.cov1, test="Chi")[2,5]

#PERMUTATING

devrank[1]=anova(m.cov1.0, m.cov1, test="Chi")[2,4]

permresults[,1]=predict(m.cov1, data.grid)

i=j=2

while (j<1001) {

index=sample(length(m.data$Asth_Count), replace=F)

m.data[,3:4]=coords[index,]

m.gam=gam(Asth_Count~ lo(Centroid_X,Centroid_Y, span=0.15)+offset(log(Population)) + SUM_OILGAS, family=poisson, data = m.data)

devrank[i]=anova(m.cov1.0, m.gam, test="Chi")[2,4]

permresults[,i]=predict(m.gam, data.grid)

i=j=j+1 }

p.adj=(1000-rank(devrank)[1])/1000

p.adj

#CREATE A LIST FOR MAPPING IN R

permrank=matrix(ncol=1000, nrow=gridlen, 0)

for (k in 1:gridlen) {

permrank[k,]=rank(permresults[k,])}

data.fit.cov1=list(grid=data.grid[,1:2], fit=exp(predict(m.cov1,data.grid)),

pointwise.permt=permrank[,1]/1000)

range(data.fit.cov1$fit)

range(data.fit.c$fit)

map2 <- colormap(data.fit.cov1, as_Spatial(datamap), contours="permrank", contours.lwd=2,mapmin=7.88, mapmax=102.15,

legend.name=" Adjusted Asthma ED Visitation(Sum_OILGAS)/10000 Population ")

#COVARIATE 2///////////////////////////////////////////////////////////////////////////////////////////////////////////////

span.aic = matrix(ncol=1,nrow=17)

for (S in 3:19) {fit=gam(Asth_Count~lo(Centroid_X,Centroid_Y,span=S*0.05)+offset(log(Population)) + Cleanups, family=poisson, data=practice)

span.aic[S-2,1]=fit$aic}# STORE THE AIC FROM THE MODEL IN THE MATRIX

sp=(which(span.aic[,1]==min(span.aic[,1]))+2)*0.05

cat(paste("The span that minimizes the AIC is ", sp,".", sep=""), fill=TRUE)

#FITTING OPTIMAL SPAN

m.cov2 = gam(Asth_Count~lo(Centroid_X,Centroid_Y,span=0.15)+offset(log(Population)) + Cleanups, family=quasipoisson, data=practice)

m.cov2.0 = gam(Asth_Count~offset(log(Population)) + Cleanups, family=poisson, data=practice)

summary.glm(m.cov2)

quantile(practice$Cleanups,c(0.25,0.75))

15.0875 - 0.5000

#14.5875

exp(1.082e-03 *14.5875)

#1.015909

#Upper 95%

exp(1.082e-03*14.5875+3.251e-04*14.5875*1.96)

#1.025396

#Lower 95%

exp(1.082e-03*14.5875-3.251e-04*14.5875*1.96)

#1.015909

#ADDING COVARIATES TO GRID

data.grid$Cleanups=median(practice$Cleanups)

#HYPOTHESIS TESTING

coords=practice[,3:4] # DATA COODINATES

m.data=practice # CREATE NEW DATASET

devrank=matrix(ncol=1, nrow=1000, 0)# DEVIANCE - GLOBAL

devrank[1]=anova(m.cov2.0, m.cov2, test="Chi")[2,4]

permresults=matrix(ncol=1000, nrow=gridlen, 0) # POINTWISE - LOCAL

permresults[,1]=predict(m.cov2, data.grid)

anova(m.cov2.0, m.cov2, test="Chi")[2,5]

#PERMUTATING

devrank[1]=anova(m.cov2.0, m.cov2, test="Chi")[2,4]

permresults[,1]=predict(m.cov2, data.grid)

i=j=2

while (j<1001) {

index=sample(length(m.data$Asth_Count), replace=F)

m.data[,3:4]=coords[index,]

m.gam=gam(Asth_Count~ lo(Centroid_X,Centroid_Y, span=0.15)+offset(log(Population)) + Cleanups, family=poisson, data = m.data)

devrank[i]=anova(m.cov2.0, m.gam, test="Chi")[2,4]

permresults[,i]=predict(m.gam, data.grid)

i=j=j+1 }

p.adj=(1000-rank(devrank)[1])/1000

p.adj

#CREATE A LIST FOR MAPPING IN R

permrank=matrix(ncol=1000, nrow=gridlen, 0)

for (k in 1:gridlen) {

permrank[k,]=rank(permresults[k,])}

data.fit.cov2=list(grid=data.grid[,1:2], fit=exp(predict(m.cov2,data.grid)),

pointwise.permt=permrank[,1]/1000)

range(data.fit.cov2$fit)

range(data.fit.c$fit)

map3 <- colormap(data.fit.cov2, as_Spatial(datamap), contours="permrank", contours.lwd=2, mapmin=7.88, mapmax=102.15,

legend.name=" Adjusted Asthma ED Visitation(Cleanups)/1000 Population ")

#COVARIATE 3///////////////////////////////////////////////////////////////////////////////////////////////////////////////

span.aic = matrix(ncol=1,nrow=17)

for (S in 3:19) {fit=gam(Asth_Count~lo(Centroid_X,Centroid_Y,span=S*0.05)+offset(log(Population)) + Solid_Waste, family=poisson, data=practice)

span.aic[S-2,1]=fit$aic}# STORE THE AIC FROM THE MODEL IN THE MATRIX

sp=(which(span.aic[,1]==min(span.aic[,1]))+2)*0.05

cat(paste("The span that minimizes the AIC is ", sp,".", sep=""), fill=TRUE)

#FITTING OPTIMAL SPAN

m.cov3 = gam(Asth_Count~lo(Centroid_X,Centroid_Y,span=0.15)+offset(log(Population)) + Solid_Waste, family=quasipoisson, data=practice)

m.cov3.0 = gam(Asth_Count~offset(log(Population)) + Solid_Waste, family=poisson, data=practice)

summary.glm(m.cov3)

quantile(practice$Solid_Waste,c(0.05,0.95))

2.5 - 0.0

#2.5

exp(2.353e-03 *2.5)

#1.0059

#Upper 95%

exp(2.353e-03*2.5+1.474e-03*2.5*1.96)

#1.013191

#Lower 95%

exp(2.353e-03*2.5-1.474e-03*2.5*1.96)

#0.9986608

#ADDING COVARIATES TO GRID

data.grid$Solid_Waste=quantile(practice$Solid_Waste, 0.05)

#HYPOTHESIS TESTING

coords=practice[,3:4] # DATA COODINATES

m.data=practice # CREATE NEW DATASET

devrank=matrix(ncol=1, nrow=1000, 0)# DEVIANCE - GLOBAL

devrank[1]=anova(m.cov3.0, m.cov3, test="Chi")[2,4]

permresults=matrix(ncol=1000, nrow=gridlen, 0) # POINTWISE - LOCAL

permresults[,1]=predict(m.cov3, data.grid)

anova(m.cov3.0, m.cov3, test="Chi")[2,5]

#PERMUTATING

devrank[1]=anova(m.cov3.0, m.cov3, test="Chi")[2,4]

permresults[,1]=predict(m.cov3, data.grid)

i=j=2

while (j<1001) {

index=sample(length(m.data$Asth_Count), replace=F)

m.data[,3:4]=coords[index,]

m.gam=gam(Asth_Count~ lo(Centroid_X,Centroid_Y, span=0.15)+offset(log(Population)) + Solid_Waste, family=poisson, data = m.data)

devrank[i]=anova(m.cov3.0, m.gam, test="Chi")[2,4]

permresults[,i]=predict(m.gam, data.grid)

i=j=j+1 }

p.adj=(1000-rank(devrank)[1])/1000

p.adj

#CREATE A LIST FOR MAPPING IN R

permrank=matrix(ncol=1000, nrow=gridlen, 0)

for (k in 1:gridlen) {

permrank[k,]=rank(permresults[k,])}

data.fit.cov3=list(grid=data.grid[,1:2], fit=exp(predict(m.cov3,data.grid)),

pointwise.permt=permrank[,1]/1000)

range(data.fit.cov3$fit)

range(data.fit.c$fit)

map4 <-colormap(data.fit.cov3, as_Spatial(datamap), contours="permrank", contours.lwd=2, mapmin=7.88, mapmax=102.15,

legend.name=" Adjusted Asthma ED Visitation(Solid_Waste)/1000 Population ")

#COVARIATE 4///////////////////////////////////////////////////////////////////////////////////////////////////////////////

span.aic = matrix(ncol=1,nrow=17)

for (S in 3:19) {fit=gam(Asth_Count~lo(Centroid_X,Centroid_Y,span=S*0.05)+offset(log(Population)) + Poverty, family=poisson, data=practice)

span.aic[S-2,1]=fit$aic}# STORE THE AIC FROM THE MODEL IN THE MATRIX

sp=(which(span.aic[,1]==min(span.aic[,1]))+2)*0.05

cat(paste("The span that minimizes the AIC is ", sp,".", sep=""), fill=TRUE)

#FITTING OPTIMAL SPAN

m.cov4 = gam(Asth_Count~lo(Centroid_X,Centroid_Y,span=0.15)+offset(log(Population)) + Poverty, family=quasipoisson, data=practice)

m.cov4.0 = gam(Asth_Count~offset(log(Population)) + Poverty, family=poisson, data=practice)

summary.glm(m.cov4)

quantile(insuranceprac$Poverty,c(0.25,0.75))

49.700 - 19.625

#30.075

exp(8.694e-03 *30.075)

#1.298841

#Upper 95%

exp(8.694e-03 *30.075+3.350e-04 *30.075*1.96)

#1.324744

#Lower 95%

exp(8.694e-03 *30.075-3.350e-04 *30.075*1.96)

#1.273444

#ADDING COVARIATES TO GRID

data.grid$Poverty=median(practice$Poverty)

#HYPOTHESIS TESTING

coords=practice[,3:4] # DATA COODINATES

m.data=practice # CREATE NEW DATASET

devrank=matrix(ncol=1, nrow=1000, 0)# DEVIANCE - GLOBAL

devrank[1]=anova(m.cov4.0, m.cov4, test="Chi")[2,4]

permresults=matrix(ncol=1000, nrow=gridlen, 0) # POINTWISE - LOCAL

permresults[,1]=predict(m.cov4, data.grid)

anova(m.cov4.0, m.cov4, test="Chi")[2,5]

#PERMUTATING

devrank[1]=anova(m.cov4.0, m.cov4, test="Chi")[2,4]

permresults[,1]=predict(m.cov4, data.grid)

i=j=2

while (j<1001) {

index=sample(length(m.data$Asth_Count), replace=F)

m.data[,3:4]=coords[index,]

m.gam=gam(Asth_Count~ lo(Centroid_X,Centroid_Y, span=0.15)+offset(log(Population)) + Poverty, family=poisson, data = m.data)

devrank[i]=anova(m.cov4.0, m.gam, test="Chi")[2,4]

permresults[,i]=predict(m.gam, data.grid)

i=j=j+1 }

p.adj=(1000-rank(devrank)[1])/1000

p.adj

#CREATE A LIST FOR MAPPING IN R

permrank=matrix(ncol=1000, nrow=gridlen, 0)

for (k in 1:gridlen) {

permrank[k,]=rank(permresults[k,])}

data.fit.cov4=list(grid=data.grid[,1:2], fit=exp(predict(m.cov4,data.grid)),

pointwise.permt=permrank[,1]/1000)

range(data.fit.cov4$fit)

range(data.fit.c$fit)

map5 <-colormap(data.fit.cov4, as_Spatial(datamap), contours="permrank", contours.lwd=2, mapmin=7.88, mapmax=102.15,

legend.name=" Adjusted Asthma ED Visitation(Poverty)/1000 Population ")

#COVARIATE 5///////////////////////////////////////////////////////////////////////////////////////////////////////////////

span.aic = matrix(ncol=1,nrow=17)

for (S in 3:19) {fit=gam(Asth_Count~lo(Centroid_X,Centroid_Y,span=S*0.05)+offset(log(Population)) + Unemployment, family=poisson, data=practice)

span.aic[S-2,1]=fit$aic}# STORE THE AIC FROM THE MODEL IN THE MATRIX

sp=(which(span.aic[,1]==min(span.aic[,1]))+2)*0.05

cat(paste("The span that minimizes the AIC is ", sp,".", sep=""), fill=TRUE)

#FITTING OPTIMAL SPAN

m.cov5 = gam(Asth_Count~lo(Centroid_X,Centroid_Y,span=0.15)+offset(log(Population)) + Unemployment, family=quasipoisson, data=practice)

m.cov5.0 = gam(Asth_Count~offset(log(Population)) + Unemployment, family=poisson, data=practice)

summary.glm(m.cov5)

quantile(insuranceprac$Unemployment,c(0.25,0.75))

7.8 - 4.0

#3.8

exp(1.644e-02 *3.8)

#1.064465

#Upper 95%

exp(1.644e-02*3.8+1.877e-03*3.8*1.96)

#1.07945

#Lower 95%

exp(1.644e-02*3.8-1.877e-03*3.8*1.96)

#1.049687

#ADDING COVARIATES TO GRID

data.grid$Unemployment=median(practice$Unemployment)

#HYPOTHESIS TESTING

coords=practice[,3:4] # DATA COODINATES

m.data=practice # CREATE NEW DATASET

devrank=matrix(ncol=1, nrow=1000, 0)# DEVIANCE - GLOBAL

devrank[1]=anova(m.cov5.0, m.cov5, test="Chi")[2,4]

permresults=matrix(ncol=1000, nrow=gridlen, 0) # POINTWISE - LOCAL

permresults[,1]=predict(m.cov5, data.grid)

anova(m.cov5.0, m.cov5, test="Chi")[2,5]

#PERMUTATING

devrank[1]=anova(m.cov5.0, m.cov5, test="Chi")[2,4]

permresults[,1]=predict(m.cov5, data.grid)

i=j=2

while (j<1001) {

index=sample(length(m.data$Asth_Count), replace=F)

m.data[,3:4]=coords[index,]

m.gam=gam(Asth_Count~ lo(Centroid_X,Centroid_Y, span=0.15)+offset(log(Population)) + Poverty, family=poisson, data = m.data)

devrank[i]=anova(m.cov5.0, m.gam, test="Chi")[2,4]

permresults[,i]=predict(m.gam, data.grid)

i=j=j+1 }

p.adj=(1000-rank(devrank)[1])/1000

p.adj

#CREATE A LIST FOR MAPPING IN R

permrank=matrix(ncol=1000, nrow=gridlen, 0)

for (k in 1:gridlen) {

permrank[k,]=rank(permresults[k,])}

data.fit.cov5=list(grid=data.grid[,1:2], fit=exp(predict(m.cov5,data.grid)),

pointwise.permt=permrank[,1]/1000)

range(data.fit.cov5$fit)

range(data.fit.c$fit)

map6 <-colormap(data.fit.cov5, as_Spatial(datamap), contours="permrank", contours.lwd=2, mapmin=7.88, mapmax=102.15,

legend.name=" Adjusted Asthma ED Visitation(Unemployment)/1000 Population ")

#SENSITIVITY ANALYSIS

calenvirorename <- calenviro %>% rename(Tract = Census.Tract)

envirotraffic2<- practice%>% left_join(calenvirorename %>% select(Tract, Traffic), by = "Tract")

Environew <- envirotraffic2%>% left_join(calenvirorename %>% select(Tract, CES.4.0.Score, Diesel.PM, PM2.5, Pesticides), by = "Tract")

gamstraffic= gam(Asth_Count~lo(Centroid_X,Centroid_Y, span=0.15) + offset(log(Population)) + SUM_OILGAS+ Cleanups + Solid_Waste + Traffic + Poverty + Unemployment, family=quasipoisson, data=envirotraffic2)

summary.glm(gamstraffic)

#COVARIATE 6 Sensitivity ///////////////////////////////////////////////////////////////////////////////////////////////////////////////

span.aic = matrix(ncol=1,nrow=17)

for (S in 3:19)

{fit=gam(Asth_Count~lo(Centroid_X,Centroid_Y,span=S*0.05)+offset(log(Population)) + CES.4.0.Score, family=poisson, data=environew) span.aic[S-2,1]=fit$aic}

# STORE THE AIC FROM THE MODEL IN THE MATRIX

sp=(which(span.aic[,1]==min(span.aic[,1]))+2)*0.05 cat(paste("The span that minimizes the AIC is ", sp,".", sep=""), fill=TRUE)

#FITTING OPTIMAL SPAN

m.cov6 = gam(Asth_Count~lo(Centroid_X,Centroid_Y,span=0.15)+offset(log(Population)) + CES.4.0.Score, family=poisson, data=environew)

m.cov6.0 = gam(Asth_Count~offset(log(Population)) + CES.4.0.Score, family=poisson, data=environew)

summary.glm(m.cov6)

quantile(environew$CES.4.0.Score,c(0.25,0.75))

51.7375 - 25.3400

#26.3975

exp( 1.643e-02 *26.3975) #1.542973*

*#Upper 95% exp( 1.643e-02*26.3975+2.818e-04*26.3975*1.96) #1.565634

#Lower 95% exp( 1.643e-02*26.3975-2.818e-04*26.3975*1.96) #1.520639

#ADDING COVARIATES TO GRID data.grid$CES.4.0.Score=median(environew$CES.4.0.Score)

#HYPOTHESIS TESTING

coords=environew[,3:4]

# DATA COODINATES m.data=environew

# CREATE NEW DATASET devrank=matrix(ncol=1, nrow=1000, 0)

# DEVIANCE - GLOBAL devrank[1]=anova(m.cov6.0, m.cov6, test="Chi")[2,4] permresults=matrix(ncol=1000, nrow=gridlen, 0) #

POINTWISE - LOCAL permresults[,1]=predict(m.cov6, data.grid)

anova(m.cov6.0, m.cov6, test="Chi")[2,5]

#PERMUTATING

devrank[1]=anova(m.cov6.0, m.cov6, test="Chi")[2,4] permresults[,1]=predict(m.cov6, data.grid)

i=j=2 while (j<1001) { index=sample(length(m.data$Asth_Count), replace=F) m.data[,3:4]=coords[index,] m.gam=gam(Asth_Count~ lo(Centroid_X,Centroid_Y, span=0.15)+offset(log(Population)) + CES.4.0.Score, family=poisson, data = m.data) devrank[i]=anova(m.cov6.0, m.gam, test="Chi")[2,4] permresults[,i]=predict(m.gam, data.grid) i=j=j+1 }

p.adj=(1000-rank(devrank)[1])/1000 p.adj

#CREATE A LIST FOR MAPPING IN R

permrank=matrix(ncol=1000, nrow=gridlen, 0) for (k in 1:gridlen) { permrank[k,]=rank(permresults[k,])}

data.fit.cov6=list(grid=data.grid[,1:2], fit=exp(predict(m.cov6,data.grid)), pointwise.permt=permrank[,1]/1000)

range(data.fit.cov6$fit)

range(data.fit.c$fit)

map7 <-colormap(data.fit.cov6, as_Spatial(datamap), contours="permrank", contours.lwd=2, mapmin=7.88, mapmax=102.15, legend.name=" Adjusted Asthma ED Visitation(CES.4.0.Score)/10000 Population ")

#COVARIATE 7 Sensitivity ///////////////////////////////////////////////////////////////////////////////////////////////////////////////

span.aic = matrix(ncol=1,nrow=17)

for (S in 3:19)

{fit=gam(Asth_Count~lo(Centroid_X,Centroid_Y,span=S*0.05)+offset(log(Population)) + Diesel.PM, family=poisson, data=environew) span.aic[S-2,1]=fit$aic}# STORE THE AIC FROM THE MODEL IN THE MATRIX

sp=(which(span.aic[,1]==min(span.aic[,1]))+2)*0.05 cat(paste("The span that minimizes the AIC is ", sp,".", sep=""), fill=TRUE)

#FITTING OPTIMAL SPAN

m.cov7 = gam(Asth_Count~lo(Centroid_X,Centroid_Y,span=0.15)+offset(log(Population)) + Diesel.PM, family=poisson, data=environew)

m.cov7.0 = gam(Asth_Count~offset(log(Population)) + Diesel.PM, family=poisson, data=environew)

summary.glm(m.cov7)

quantile(environew$Diesel.PM,c(0.25,0.75)) 0.39375 - 0.11225 #0.28

exp(4.838e-02 *0.28) #1.013639

#Upper 95% exp(4.838e-02*0.28+1.677e-02*0.28*1.96) #1.023011*

*#Lower 95% exp(4.838e-02*0.28-1.677e-02*0.28*1.96) #1.004352

#ADDING COVARIATES TO GRID data.grid$Diesel.PM=median(environew$Diesel.PM)

#HYPOTHESIS TESTING

coords=environew[,3:4] # DATA COODINATES m.data=environew # CREATE NEW DATASET devrank=matrix(ncol=1, nrow=1000, 0)# DEVIANCE - GLOBAL devrank[1]=anova(m.cov7.0, m.cov7, test="Chi")[2,4] permresults=matrix(ncol=1000, nrow=gridlen, 0) # POINTWISE - LOCAL permresults[,1]=predict(m.cov7, data.grid)

anova(m.cov7.0, m.cov7, test="Chi")[2,5]

#PERMUTATING

devrank[1]=anova(m.cov7.0, m.cov7, test="Chi")[2,4] permresults[,1]=predict(m.cov7, data.grid)

i=j=2 while (j<1001) { index=sample(length(m.data$Asth_Count), replace=F) m.data[,3:4]=coords[index,] m.gam=gam(Asth_Count~ lo(Centroid_X,Centroid_Y, span=0.15)+offset(log(Population)) + Diesel.PM, family=poisson, data = m.data) devrank[i]=anova(m.cov7.0, m.gam, test="Chi")[2,4] permresults[,i]=predict(m.gam, data.grid) i=j=j+1 }

p.adj=(1000-rank(devrank)[1])/1000 p.adj

#CREATE A LIST FOR MAPPING IN R

permrank=matrix(ncol=1000, nrow=gridlen, 0) for (k in 1:gridlen) { permrank[k,]=rank(permresults[k,])}

data.fit.cov7=list(grid=data.grid[,1:2], fit=exp(predict(m.cov7,data.grid)), pointwise.permt=permrank[,1]/1000)

range(data.fit.cov7$fit)

range(data.fit.c$fit)

map8 <-colormap(data.fit.cov7, as_Spatial(datamap), contours="permrank", contours.lwd=2, mapmin=7.88, mapmax=102.15, legend.name=" Adjusted Asthma ED Visitation(Diesel.PM)/10000 Population ")

#COVARIATE 8 Sensitivity ///////////////////////////////////////////////////////////////////////////////////////////////////////////////

span.aic = matrix(ncol=1,nrow=17)

or (S in 3:19)

{fit=gam(Asth_Count~lo(Centroid_X,Centroid_Y,span=S*0.05)+offset(log(Population)) + PM2.5, family=poisson, data=environew) span.aic[S-2,1]=fit$aic}

# STORE THE AIC FROM THE MODEL IN THE MATRIX

sp=(which(span.aic[,1]==min(span.aic[,1]))+2)*0.05 cat(paste("The span that minimizes the AIC is ", sp,".", sep=""), fill=TRUE)

#FITTING OPTIMAL SPAN

m.cov8 = gam(Asth_Count~lo(Centroid_X,Centroid_Y,span=0.15)+offset(log(Population)) + PM2.5, family=poisson, data=environew)

m.cov8.0 = gam(Asth_Count~offset(log(Population)) + PM2.5, family=poisson, data=environew)

summary.glm(m.cov8)

quantile(environew$PM2.5,c(0.25,0.75)) 12.08560 - 11.68011 #0.40549

exp(2.007e-01 *0.40549) #1.084785*

*#Upper 95% exp(2.007e-01*0.40549+1.081e-02*0.40549*1.96) #1.094145

#Lower 95% exp(2.007e-01*0.40549-1.081e-02*0.40549*1.96) #1.075505

#ADDING COVARIATES TO GRID data.grid$PM2.5=median(environew$PM2.5)

#HYPOTHESIS TESTING

coords=environew[,3:4] # DATA COODINATES m.data=environew # CREATE NEW DATASET devrank=matrix(ncol=1, nrow=1000, 0)# DEVIANCE - GLOBAL devrank[1]=anova(m.cov8.0, m.cov8, test="Chi")[2,4] permresults=matrix(ncol=1000, nrow=gridlen, 0) # POINTWISE - LOCAL permresults[,1]=predict(m.cov8, data.grid)

anova(m.cov8.0, m.cov8, test="Chi")[2,5]

#PERMUTATING

devrank[1]=anova(m.cov8.0, m.cov8, test="Chi")[2,4] permresults[,1]=predict(m.cov8, data.grid)

i=j=2 while (j<1001) { index=sample(length(m.data$Asth_Count), replace=F) m.data[,3:4]=coords[index,] m.gam=gam(Asth_Count~ lo(Centroid_X,Centroid_Y, span=0.15)+offset(log(Population)) + PM2.5, family=poisson, data = m.data) devrank[i]=anova(m.cov8.0, m.gam, test="Chi")[2,4] permresults[,i]=predict(m.gam, data.grid) i=j=j+1 }

p.adj=(1000-rank(devrank)[1])/1000 p.adj

#CREATE A LIST FOR MAPPING IN R

permrank=matrix(ncol=1000, nrow=gridlen, 0) for (k in 1:gridlen) { permrank[k,]=rank(permresults[k,])}

data.fit.cov8=list(grid=data.grid[,1:2], fit=exp(predict(m.cov8,data.grid)), pointwise.permt=permrank[,1]/1000)

range(data.fit.cov8$fit)

range(data.fit.c$fit)

map8 <-colormap(data.fit.cov8, as_Spatial(datamap), contours="permrank", contours.lwd=2, mapmin=7.88, mapmax=102.15, legend.name=" Adjusted Asthma ED Visitation(PM2.5)/10000 Population ")

#COVARIATE 9 Sensitivity ///////////////////////////////////////////////////////////////////////////////////////////////////////////////

span.aic = matrix(ncol=1,nrow=17)

for (S in 3:19)

{fit=gam(Asth_Count~lo(Centroid_X,Centroid_Y,span=S*0.05)+offset(log(Population)) + Pesticides, family=poisson, data=environew) span.aic[S-2,1]=fit$aic}

# STORE THE AIC FROM THE MODEL IN THE MATRIX

sp=(which(span.aic[,1]==min(span.aic[,1]))+2)*0.05 cat(paste("The span that minimizes the AIC is ", sp,".", sep=""), fill=TRUE)

#FITTING OPTIMAL SPAN

m.cov9 = gam(Asth_Count~lo(Centroid_X,Centroid_Y,span=0.15)+offset(log(Population)) + Pesticides, family=poisson, data=environew)

m.cov9.0 = gam(Asth_Count~offset(log(Population)) + Pesticides, family=poisson, data=environew)

summary.glm(m.cov9)

quantile(environew$Pesticides,c(0.25,0.75)) 0 - 0 #0

exp(-1.962e-03) #0.998

#Upper 95% exp(-1.962e-03*+9.654e-04*1.96) #0.999*

*#Lower 95% exp(-1.962e-03*-9.654e-04*1.96) #1.00

#ADDING COVARIATES TO GRID data.grid$Pesticides=median(environew$Pesticides)

#HYPOTHESIS TESTING

coords=environew[,3:4] # DATA COODINATES m.data=environew # CREATE NEW DATASET devrank=matrix(ncol=1, nrow=1000, 0)# DEVIANCE - GLOBAL devrank[1]=anova(m.cov9.0, m.cov9, test="Chi")[2,4] permresults=matrix(ncol=1000, nrow=gridlen, 0) # POINTWISE - LOCAL permresults[,1]=predict(m.cov9, data.grid)

anova(m.cov9.0, m.cov9, test="Chi")[2,5]

#PERMUTATING

devrank[1]=anova(m.cov9.0, m.cov9, test="Chi")[2,4] permresults[,1]=predict(m.cov9, data.grid)

i=j=2 while (j<1001) { index=sample(length(m.data$Asth_Count), replace=F) m.data[,3:4]=coords[index,] m.gam=gam(Asth_Count~ lo(Centroid_X,Centroid_Y, span=0.15)+offset(log(Population)) + Pesticides, family=poisson, data = m.data) devrank[i]=anova(m.cov9.0, m.gam, test="Chi")[2,4] permresults[,i]=predict(m.gam, data.grid) i=j=j+1 }

p.adj=(1000-rank(devrank)[1])/1000 p.adj

#CREATE A LIST FOR MAPPING IN R

permrank=matrix(ncol=1000, nrow=gridlen, 0) for (k in 1:gridlen) { permrank[k,]=rank(permresults[k,])}

data.fit.cov9=list(grid=data.grid[,1:2], fit=exp(predict(m.cov9,data.grid)), pointwise.permt=permrank[,1]/1000)

range(data.fit.cov9$fit)

range(data.fit.c$fit)

map8 <-colormap(data.fit.cov9, as_Spatial(datamap), contours="permrank", contours.lwd=2, mapmin=7.88, mapmax=102.15, legend.name=" Adjusted Asthma ED Visitation(Pesticides)/10000 Population ")

#////////////////Adjusted Model (Sensitivity)//////////////////////////////

#ADJUSTED ANALYSIS

span.aic = matrix(ncol=1,nrow=17) # CREATES A MATRIX TO STORE AICS

for (S in 3:19) {

fit=gam(Asth_Count~ lo(Centroid_X,Centroid_Y, span=S*0.05)+offset(log(Population)) + SUM_OILGAS + Poverty + Unemployment + Solid_Waste + Cleanups + CES.4.0.Score + Diesel.PM + PM2.5 + Pesticides, family=poisson, data = environew)

span.aic[S-2,1]=fit$aic

cat(paste("The AIC for span=", S*0.05," is ", fit$aic,".", sep=""),fill=TRUE)}

sp=(which(span.aic[,1]==min(span.aic[,1]))+2)*0.05

cat(paste("The span that minimizes the AIC is ", sp,".", sep=""), fill=TRUE)

#FITTING OPTIMAL SPAN

m.adjs=gam(Asth_Count~ lo(Centroid_X,Centroid_Y, span=0.15)+offset(log(Population)) + SUM_OILGAS + Poverty + Unemployment + Solid_Waste + Cleanups + CES.4.0.Score + Diesel.PM + PM2.5 + Pesticides, family=poisson, data = environew)

summary.glm(m.adjs)

#FITTING WITHOUT SMOOTHING TERM

m.adjs.0=gam(Asth_Count~offset(log(Population)) + SUM_OILGAS + Poverty + Unemployment + Solid_Waste + Cleanups + CES.4.0.Score + Diesel.PM + PM2.5 + Pesticides, family=poisson, data =environew)

#ADDING COVARIATES TO GRID

data.grid$Poverty=median(environew$Poverty)

data.grid$Unemployment=median(environew$Unemployment)

data.grid$Solid_Waste = median(environew$Solid_Waste)

data.grid$Cleanups = median(environew$Cleanups)

data.grid$SUM_OILGAS = median(environew$SUM_OILGAS)

data.grid$CES.4.0.Score=median(environew$CES.4.0.Score)

data.grid$Diesel.PM=median(environew$Diesel.PM)

data.grid$PM2.5 = median(environew$PM2.5)

data.grid$Pesticides = median(environew$Pesticides)

#HYPOTHESIS TESTING

anova(m.adjs.0, m.adjs, test="Chi")[2,5]

#PERMUTATING

devrank[1]=anova(m.adjs.0, m.adjs, test="Chi")[2,4]

permresults[,1]=predict(m.adjs, data.grid)

i=j=2

while (j<1001) {

index=sample(length(m.data$Asth_Count), replace=F)

m.data[,3:4]=coords[index,]

m.gam=gam(Asth_Count~ lo(Centroid_X,Centroid_Y, span=0.15)+offset(log(Population)) + SUM_OILGAS + Poverty + Unemployment + Solid_Waste + Cleanups + CES.4.0.Score + Diesel.PM + PM2.5 + Pesticides , family=poisson, data = m.data)

devrank[i]=anova(m.adjs.0, m.gam, test="Chi")[2,4]

permresults[,i]=predict(m.gam, data.grid)

i=j=j+1 }

#GLOBAL SIGNIFICANCE

p.adj=(1000-rank(devrank)[1])/1000

p.adj

#CREATE A LIST FOR MAPPING IN R

permrank=matrix(ncol=1000, nrow=gridlen, 0)

for (k in 1:gridlen) {

permrank[k,]=rank(permresults[k,])}

data.fit.as=list(grid=data.grid[,1:2], fit=exp(predict(m.adjs,data.grid)),

pointwise.permt=permrank[,1]/1000)

range(data.fit.as$fit)

range(data.fit.c$fit)

#WITH MIN AND MAX

map10 <- colormap(data.fit.as, as_Spatial(datamap), contours="permrank", contours.lwd=2, mapmin=7.875047, mapmax=102.149586, legend.name=" Adjusted Asthma ED Visitation/10000 Population (Sens) ")

#WITHOUT MAPMIN AND MAPMAX

colormap(data.fit.as, as_Spatial(datamap), contours="permrank", contours.lwd=2,legend.name=" Adjusted Asthma ED Visitation/10000 Population (Sens)")

#////////////////Adjusted Model//////////////////////////////

#ADJUSTED ANALYSIS

span.aic = matrix(ncol=1,nrow=17) # CREATES A MATRIX TO STORE AICS

for (S in 3:19) {

fit=gam(Asth_Count~ lo(Centroid_X,Centroid_Y, span=S*0.05)+offset(log(Population)) + SUM_OILGAS + Poverty + Unemployment + Solid_Waste + Cleanups, family=poisson, data = practice)

span.aic[S-2,1]=fit$aic

cat(paste("The AIC for span=", S*0.05," is ", fit$aic,".", sep=""),fill=TRUE)}

sp=(which(span.aic[,1]==min(span.aic[,1]))+2)*0.05

cat(paste("The span that minimizes the AIC is ", sp,".", sep=""), fill=TRUE)

#FITTING OPTIMAL SPAN

m.adj=gam(Asth_Count~ lo(Centroid_X,Centroid_Y, span=0.15)+offset(log(Population)) + SUM_OILGAS + Poverty + Unemployment + Solid_Waste + Cleanups, family=poisson, data = practice)

summary.glm(m.adj)

#FITTING WITHOUT SMOOTHING TERM

m.adj.0=gam(Asth_Count~offset(log(Population)) + SUM_OILGAS + Poverty + Unemployment + Solid_Waste + Cleanups, family=poisson, data = practice)

#ADDING COVARIATES TO GRID

data.grid$Poverty=median(practice$Poverty)

data.grid$Unemployment=median(practice$Unemployment)

data.grid$Solid_Waste = median(practice$Solid_Waste)

data.grid$Cleanups = median(practice$Cleanups)

data.grid$SUM_OILGAS = median(practice$SUM_OILGAS)

#HYPOTHESIS TESTING

anova(m.adj.0, m.adj, test="Chi")[2,5]

#PERMUTATING

devrank[1]=anova(m.adj.0, m.adj, test="Chi")[2,4]

permresults[,1]=predict(m.adj, data.grid)

i=j=2

while (j<1001) {

index=sample(length(m.data$Asth_Count), replace=F)

m.data[,3:4]=coords[index,]

m.gam=gam(Asth_Count~ lo(Centroid_X,Centroid_Y, span=0.15)+offset(log(Population)) + SUM_OILGAS + Poverty + Unemployment + Solid_Waste + Cleanups , family=poisson, data = m.data)

devrank[i]=anova(m.adj.0, m.gam, test="Chi")[2,4]

permresults[,i]=predict(m.gam, data.grid)

i=j=j+1 }

#GLOBAL SIGNIFICANCE

p.adj=(1000-rank(devrank)[1])/1000

p.adj

#CREATE A LIST FOR MAPPING IN R

permrank=matrix(ncol=1000, nrow=gridlen, 0)

for (k in 1:gridlen) {

permrank[k,]=rank(permresults[k,])}

data.fit.a=list(grid=data.grid[,1:2], fit=exp(predict(m.adj,data.grid)),

pointwise.permt=permrank[,1]/1000)

range(data.fit.a$fit)

range(data.fit.c$fit)

#WITH MIN AND MAX

map7 <- colormap(data.fit.a, as_Spatial(datamap), contours="permrank", contours.lwd=2, mapmin=7.875047, mapmax=102.149586, legend.name=" Adjusted Asthma ED Visitation/10000 Population ")

#WITHOUT MAPMIN AND MAPMAX

colormap(data.fit.a, as_Spatial(datamap), contours="permrank", contours.lwd=2,legend.name=" Adjusted Asthma ED Visitation/10000 Population ")

#///////////////////////////////////////////////////////////////////////////////////////////////////////

#Selected Model (Quasipoisson)

gamqp = gam(Asth_Count~lo(Centroid_X,Centroid_Y, span=0.15) + offset(log(Population))+

SUM_OILGAS + Cleanups + Solid_Waste + Poverty + Unemployment,

family=quasipoisson, data=practice)

summary.glm(gamqp)

quantile(insuranceprac$SUM_OILGAS,c(0.25,0.75))

3.5 - 0

#3.5

exp(-2.808e-05 *3.5)

#0.9999017

#Upper 95%

exp(-2.808e-05*3.5+2.594e-05*3.5*1.96)

#1.00008

#Lower 95%

exp(-2.808e-05*3.5-2.594e-05*3.5*1.96)

#0.9997238

quantile(insuranceprac$Cleanups,c(0.25,0.75))

15.0875 - 0.5000

#14.5875

exp(5.718e-04 *14.5875)

#1.008376

#Upper 95%

exp(5.718e-04*14.5875+3.435e-04*14.5875*1.96)

#1.018328

#Lower 95%

exp(5.718e-04*14.5875-3.435e-04*14.5875*1.96)

#0.998521

quantile(insuranceprac$Solid_Waste,c(0.25,0.75))

2.5 - 0.0

#2.5

exp(3.132e-03 *2.5)

#1.007861

#Upper 95%

exp(3.132e-03*2.5+1.517e-03*2.5*1.96)

#1.01538

#Lower 95%

exp(3.132e-03*2.5-1.517e-03*2.5*1.96)

#0.1.000397

quantile(insuranceprac$Poverty,c(0.25,0.75))

49.700 - 19.625

#30.075

exp(8.394e-03 *30.075)

#1.287175

#Upper 95%

exp(8.394e-03*30.075+3.763e-04*30.075*1.96)

#1.316045

#Lower 95%

exp(8.394e-03*30.075-3.763e-04*30.075*1.96)

#1.258937

quantile(insuranceprac$Unemployment,c(0.25,0.75))

7.8 - 4.0

#3.8

exp(4.331e-03 *3.8)

#1.016594

#Upper 95%

exp(4.331e-03*3.8+1.998e-03*3.8*1.96)

#1.031835

#Lower 95%

exp(4.331e-03*3.8-1.998e-03*3.8*1.96)

#1.001578

#SENSITIVITY ANALYSIS

gamsensitivity= gam(Asth_Count~lo(Centroid_X,Centroid_Y, span=0.15) + offset(log(Population)) + SUM_OILGAS+ Cleanups + Solid_Waste + Poverty + Unemployment + Diesel.PM + PM2.5 + Pesticides, family=quasipoisson, data=environew)

summary.glm(gamsensitivity)

#////////SENSITIVITY ANALYSIS///////

quantile(envirotraffic2$Traffic,c(0.25,0.75),na.rm = T)

1860.3778 - 788.8117 #1071.566

exp(-2.810e-05*1071.566) #0.9703378 #upper95% exp(-2.810e-05*1071.566+6.426e-06*1071.566*1.96) #0.9835226 #Lower95% exp(-2.810e-05*1071.566-6.426e-06*1071.566*1.96) #0.9573298

#/ quantile(environew$SUM_OILGAS,c(0.25,0.75))

3.5 - 0 #3.5

exp( -2.299e-05 *3.5) #0.9999195 #Upper 95%*

*exp( -2.299e-05*3.5+2.584e-05*3.5*1.96) #1.000097 #Lower 95%

exp( -2.299e-05*3.5-2.584e-05*3.5*1.96) #0.9997423

quantile(environew$Cleanups,c(0.25,0.75))

15.0875 - 0.5000 #14.5875

exp(6.225e-04 *14.5875) #1.009122 #Upper 95% exp(6.225e-04*14.5875+6.225e-04*14.5875*1.96) #1.027243 #Lower 95% exp(6.225e-04*14.5875-6.225e-04*14.5875*1.96) #0.9913204

quantile(environew$Solid_Waste,c(0.25,0.75))

2.5 - 0.0 #2.5

exp(3.103e-03 *2.5) #1.007788 #Upper 95%*

*exp(3.103e-03*2.5+ 1.522e-03*2.5*1.96) #1.015332 #Lower 95%

exp(3.103e-03*2.5- 1.522e-03*2.5*1.96) #1.0003

quantile(environew$Poverty,c(0.25,0.75))

49.700 - 19.625 #30.075

exp(8.237e-03 *30.075) #1.281111 #Upper 95% exp(8.237e-03*30.075+3.949e-04*30.075*1.96) #1.311283 #Lower 95% exp(8.237e-03*30.075-3.949e-04*30.075*1.96) #1.251634

quantile(environew$Unemployment,c(0.25,0.75))

7.8 - 4.0 #3.8

exp(4.599e-03 *3.8) #1.01763 #Upper 95%*

*exp(4.599e-03*3.8+ 1.988e-03*3.8*1.96) #1.03281 #Lower 95%

exp(4.599e-03*3.8- 1.988e-03*3.8*1.96) #1.002673

quantile(environew$CES.4.0.Score,c(0.25,0.75))

51.74 - 25.34 #26.4

exp(2.129e-02 *26.4) #1.754276 #Upper 95%*

*exp(2.129e-02*26.4+6.277e-04*26.4*1.96) #1.812189 #Lower 95% exp(2.129e-02*26.4-6.277e-04*26.4*1.96) #1.698213

quantile(environew$Diesel.PM,c(0.25,0.75)) 0.39 - 0.11 #0.28

exp(-5.212e-02 *0.28) *#*0.9855124 #Upper 95%

exp(-5.212e-02*0.28+2.404e-02*0.28*1.96) #0.9986005 #Lower 95%*

*exp(-5.212e-02*0.28-2.404e-02*0.28*1.96) #0.9725957

quantile(environew$PM2.5,c(0.25,0.75))

12.08 - 11.68 #3.8

exp(1.499e-01 *0.4) #1.061794 #Upper 95%*

*exp(1.499e-01*0.4+ 1.527e-02*0.4*1.96) #1.074582 #Lower 95%

exp(1.499e-01*0.4- 1.527e-02*0.4*1.96) #1.049158

quantile(environew$Pesticides,c(0.25,0.75)) 0 - 0 #0

exp(-1.855e-03) #0.9981467 #Upper 95%

exp(-1.855e-03+1.301e-03*1.96) #1.000695 #Lower 95%*

*exp(-1.855e-03+-1.301e-03*1.96) #0.9956047

#variance-mean relationship

mu_hat <- fitted(gamquasi)

resid_sq <- residuals(gamquasi, type = "pearson")^2

plot(mu_hat, resid_sq,

xlab = "Fitted Mean (mu)",

ylab = "Squared Pearson Residuals",

main = "Variance vs. Mean (GAM Quasi-Poisson)")

abline(lm(resid_sq ~ mu_hat), col = "red")

#Exploratory Analyses of Environmental indicators

practice$log_Solid_Waste <- log(practice$Solid_Waste + 1)

practice$log_cleanups <- log(practice$Cleanups + 1)

practice$log_oil <- log(practice$SUM_OILGAS + 1)

smoothloggamquasi = gam(Asth_Count~lo(Centroid_X, Centroid_Y, span = 0.15) + offset(log(Population))+

lo(log_oil, span=0.25) + lo(log_cleanups, span=0.25) + lo(log_Solid_Waste, span = 0.50) + Poverty + Unemployment,

family = quasipoisson(link = "log"), data = practice)

summary.glm(smoothloggamquasi)

#////////////////////////////////////////////////////////////////

span.aic = matrix(ncol=1,nrow=19)

for (S in 1:19) {

fit=gam(Asth_Count~lo(Centroid_X,Centroid_Y, span=0.15) + lo(log_oil,span=S*0.05) + offset(log(Population)) + Poverty + Unemployment, family=poisson, data=practice)

span.aic[S,1]=fit$aic

cat(paste("The AIC for span=",format(S*0.05,nsmall=2)," is ",format(fit$aic,nsmall=2),".", sep=""),fill=TRUE)

}

spc=(which(span.aic[,1]==min(span.aic[,1])))*0.05

cat(paste("The span that minimizes the AIC is ",format(spc,nsmall=2),".", sep=""),fill=TRUE)

# Fit model

m.oil=gam(Asth_Count~ lo(log_oil, span=0.25) + offset(log(Population)), family=poisson, data=practice)

summary.glm(m.oil)

oil.grid=data.frame(log_oil=seq(min(practice$log_oil), max(practice$log_oil), length.out = 10000))

oil.grid$Population=10000

pred.oil=exp(predict(m.oil,oil.grid))

plot(oil.grid$log_oil,pred.oil,type ='l')

oil.grid$Centroid_X=median(practice$Centroid_X)

oil.grid$Centroid_Y=median(practice$Centroid_Y)

oil.grid$log_cleanups=median(practice$log_cleanups)

oil.grid$log_Solid_Waste=median(practice$log_Solid_Waste )

oil.grid$Poverty=median(practice$Poverty)

oil.grid$Unemployment=median(practice$Unemployment)

pred.oil=exp(predict(smoothloggamquasi,oil.grid))

plot(oil.grid$log_oil,pred.oil, type ="l")

#confidence intervals

lines(oil.grid$log_oil,exp(predict(smoothloggamquasi,oil.grid)-1.96*(3.657e-03)))

lines(oil.grid$log_oil,exp(predict(smoothloggamquasi,oil.grid)+1.96*(3.657e-03)))

#////////////////////////////////////////////////////////////////////////////////////

span.aic = matrix(ncol=1,nrow=19)

for (S in 1:19) {

fit=gam(Asth_Count~lo(Centroid_X,Centroid_Y, span=0.15) + lo(log_cleanups,span=S*0.05) + offset(log(Population)) + Poverty + Unemployment, family=poisson, data=practice)

span.aic[S,1]=fit$aic

cat(paste("The AIC for span=",format(S*0.05,nsmall=2)," is ",format(fit$aic,nsmall=2),".", sep=""),fill=TRUE)

}

spc=(which(span.aic[,1]==min(span.aic[,1])))*0.05

cat(paste("The span that minimizes the AIC is ",format(spc,nsmall=2),".", sep=""),fill=TRUE)

# Fit model

m.cleanups=gam(Asth_Count~ lo(log_cleanups, span=0.25) + offset(log(Population)), family=poisson, data=practice)

summary.glm(m.cleanups)

cleanups.grid=data.frame(log_cleanups=seq(min(practice$log_cleanups), max(practice$log_cleanups), length.out = 10000))

cleanups.grid$Population=10000

pred.cleanups=exp(predict(m.cleanups,cleanups.grid))

plot(cleanups.grid$log_cleanups,pred.cleanups,type ='l')

cleanups.grid$Centroid_X=median(practice$Centroid_X)

cleanups.grid$Centroid_Y=median(practice$Centroid_Y)

cleanups.grid$log_oil=median(practice$log_oil)

cleanups.grid$log_Solid_Waste=median(practice$log_Solid_Waste )

cleanups.grid$Poverty=median(practice$Poverty)

cleanups.grid$Unemployment=median(practice$Unemployment)

pred.cleanups=exp(predict(smoothloggamquasi,cleanups.grid))

plot(cleanups.grid$log_cleanups,pred.cleanups, type ="l")

#//////////////////////////////////////////////////////////////////////////////////////////

span.aic = matrix(ncol=1,nrow=19)

for (S in 1:19) {

fit=gam(Asth_Count~lo(Centroid_X,Centroid_Y, span=0.15) + lo(log_Solid_Waste,span=S*0.05) + offset(log(Population)) + Poverty + Unemployment, family=poisson, data=practice)

span.aic[S,1]=fit$aic

cat(paste("The AIC for span=",format(S*0.05,nsmall=2)," is ",format(fit$aic,nsmall=2),".", sep=""),fill=TRUE)

}

spc=(which(span.aic[,1]==min(span.aic[,1])))*0.05

cat(paste("The span that minimizes the AIC is ",format(spc,nsmall=2),".", sep=""),fill=TRUE)

# Fit model

m.waste=gam(Asth_Count~ lo(log_Solid_Waste, span=0.50) + offset(log(Population)), family=poisson, data=practice)

summary(m.waste)

waste.grid=data.frame(log_Solid_Waste=seq(min(practice$log_Solid_Waste), max(practice$log_Solid_Waste), length.out = 100))

waste.grid$Population=10000

pred.waste=exp(predict(m.waste,waste.grid))

plot(waste.grid$log_Solid_Waste, pred.waste, type = "l")

waste.grid$Centroid_X=median(practice$Centroid_X)

waste.grid$Centroid_Y=median(practice$Centroid_Y)

waste.grid$log_cleanups=median(practice$log_cleanups)

waste.grid$log_oil=median(practice$log_oil)

waste.grid$Poverty=median(practice$Poverty)

waste.grid$Unemployment=median(practice$Unemployment)

pred.waste=(predict(smoothloggamquasi,waste.grid))

plot(waste.grid$log_Solid_Waste, pred.waste, type ="l")

reference <- median(pred.waste)

pred <- predict(m.waste,waste.grid)

rr <- exp(pred - pred[reference])

plot(waste.grid$log_Solid_Waste, rr, type = "l",

xlab = "Log Solid Waste", ylab = "Risk Ratio",

main = "Risk Ratio Curve")

set.seed(333)

rug(practice$log_Solid_Waste)

#////////////////////////////////////////////////////////////////////////////////////

m.waste=gam(Asth_Count~ lo(log_Solid_Waste,span=0.50) +

offset(log(Population)), family=quasipoisson, data=practice)

#BOOTSTRAPPING

gridlen=length(waste.grid[,1])

sw=practice$log_Solid_Waste

m.data=practice

bootresults <- matrix(NA, nrow = gridlen, ncol = 2000)

i=j=1

while (j<2001) {

index <- sample(nrow(m.data), replace = TRUE)

boot_data <- m.data[index, ]

m.gam=gam(Asth_Count~ lo(log_Solid_Waste,span=0.50) +

offset(log(Population)), family=quasipoisson, data = boot_data)

bootresults[,i]=predict(m.gam, waste.grid)

i=j=j+1}

ref_index <- which.min(abs(waste.grid$log_Solid_Waste - median(practice$log_Solid_Waste)))

pred.waste <- predict(m.waste, waste.grid)

ref_val <- pred.waste[ref_index]

CIs=matrix(ncol=2, nrow=gridlen, 0)

for (k in 1:gridlen) {

log_rr_boot <- bootresults[k, ] - bootresults[ref_index, ]

log_CIs <- quantile(log_rr_boot, c(0.025, 0.975), na.rm = TRUE)

CIs[k, 1:2] <- exp(log_CIs)}

rr_main <- exp(pred.waste - ref_val)

plot(waste.grid$log_Solid_Waste,rr_main,type = 'l',

xlab = "Log Solid Waste", ylab = "Risk Ratio", main = "Risk Ratio with 95% CI")

lines(waste.grid$log_Solid_Waste, CIs[,1], col = "red", lty = 2) # lower CI

lines(waste.grid$log_Solid_Waste, CIs[,2], col = "red", lty = 2) # upper CI

rug(practice$log_Solid_Waste)

#///////////////////////////////////////////////////////////////////////////////////////////////////

install.packages(psych)

library(psych)

describeBy(practice, group = practice$quartile_group)

summary(practice)

practice <- practice %>%

mutate(quartile_group = ntile(Asth_Count, 4))

summary(practice$quartile_group)

tapply(practice, practice$quartile_group, summary)

33.00 - 12.45

6.800 - 3.300

45.10 - 18.50

7.300 - 3.800

19.0000 - 0.9125

51.80 - 25.90

8.100 - 4.425

16.788 - 1.700

57.50 - 34.05

8.900 - 4.525
